# Multiplex Gene Tagging with CRISPR-Cas9 for Live-Cell Microscopy and Application to Study the Role of SARS-CoV-2 Proteins in Autophagy, Mitochondrial Dynamics, and Cell Growth

**DOI:** 10.1089/crispr.2021.0041

**Published:** 2021-12-16

**Authors:** Oscar Perez-Leal, Jonathon Nixon-Abell, Carlos A. Barrero, John C. Gordon, James Oesterling, Mario C. Rico

**Affiliations:** ^1^ Department of Pharmaceutical Sciences, Moulder Center for Drug Discovery, School of Pharmacy, Temple University, Philadelphia, Pennsylvania, USA; ^2^ Janelia Research Campus, Howard Hughes Medical Institute (HHMI), Ashburn, Virginia, USA; ^3^ Flow Cytometry and Cell Sorting Facility, Fox Chase Cancer Center, Philadelphia, Pennsylvania, USA.

## Abstract

The lack of efficient tools to label multiple endogenous targets in cell lines without staining or fixation has limited our ability to track physiological and pathological changes in cells over time via live-cell studies. Here, we outline the FAST-HDR vector system to be used in combination with CRISPR-Cas9 to allow visual live-cell studies of up to three endogenous proteins within the same cell line. Our approach utilizes a novel set of advanced donor plasmids for homology-directed repair and a streamlined workflow optimized for microscopy-based cell screening to create genetically modified cell lines that do not require staining or fixation to accommodate microscopy-based studies. We validated this new methodology by developing two advanced cell lines with three fluorescent-labeled endogenous proteins that support high-content imaging without using antibodies or exogenous staining. We applied this technology to study seven severe acute respiratory syndrome coronavirus 2 (SARS-CoV-2/COVID-19) viral proteins to understand better their effects on autophagy, mitochondrial dynamics, and cell growth. Using these two cell lines, we were able to identify the protein ORF3a successfully as a potent inhibitor of autophagy, inducer of mitochondrial relocalization, and a growth inhibitor, which highlights the effectiveness of live-cell studies using this technology.

## Introduction

Microscopy-based screening techniques provide powerful visualization tools for studying physiological or pathological changes in culture cells in a high-throughput manner.^1,2^ Common areas of study include identification of cell growth, viability, shape, and size, and functionality of cellular structures. Scientists can also perform more detailed observations, including changes in localization or concentration of proteins of interest under different conditions.^3,4^ Very often, image acquisition requires cellular fixation followed by the use of fluorescent staining agents and antibodies for detecting targets of interest, thus limiting the study of dynamic subcellular changes to point-in-time analysis.^5,6^

The recent discovery and development of CRISPR-Cas9 for genome editing has enabled protein tagging via genetically modifying the endogenous genes to include molecular tags, such as fluorescent proteins, which can be detected through microscopy-based techniques without staining or fixing, and under physiological conditions.^7^ To create such cell lines, a donor DNA template containing the genetic information must first be created then inserted (typically using homology-directed repair [HDR]^8^) after cutting the DNA via a double-strand break (DSB) with CRISPR-Cas9 endonucleases.

The donor templates used to promote HDR are large plasmids containing homologous recombination arms for the 5′ and 3′ ends of the cut site.^9^ Currently, access to plasmid backbones to develop HDR donor templates is limited to a few commercial plasmids or a small selection of plasmids available in a nonprofit plasmid repository.^10,11^ These plasmids typically only allow tagging with one specific protein marker and also limit the selection of modified cell lines to only one type of eukaryotic resistance gene.^10,11^ Therefore, most researchers interested in tagging an endogenous gene follow guidelines to build their own donor template plasmids.^12^

To generate these templates, the assembly of a recombination cassette containing four main elements are required, each of which are assembled separately in a multi-step process that can take up to 3 weeks to complete for every target gene.^7^ We proposed that redesigning the donor template plasmid to facilitate the construction of advanced homologous recombination plasmids could circumvent the above limitations while simplifying and accelerating the overall cell-line creation process. The ideal backbone template would allow the insertion of any protein tag of interest for at least three tags in the same cell line (multiplexing) to facilitate high-content imaging analysis. It should also simplify the cell selection process and ideally shorten the overall cell line creation time.

Here, we report the development of the FAST-HDR vector system that combines a novel set of advanced donor plasmids for HDR in a streamlined workflow that simplifies and enhances the process for developing next-generation cell lines with tagged endogenous genes. We designed the FAST-HDR system as a series of backbone plasmids that facilitate the creation of donor templates for inserting multiple types of large labeling tags.

Each backbone plasmid, with a specific labeling tag, allows the incorporation of both the 5′ and 3′ recombination arms in a single enzymatic reaction, thus avoiding the traditional multi-step assembly process. More importantly, this system enhances the complexity of the cell models that can be generated by introducing multiplexing tagging of up to three genes. That is, these gene-edited cell lines require neither staining nor fixing to perform visual analysis of three native proteins. Furthermore, the construction of these plasmids is achieved in a single-step reaction that can be completed in only 1 day versus ∼21 days with current methodologies,^7^ while also achieving additional benefits downstream that further accelerate the overall cell-line creation process to <15 days compared to ∼8 weeks using current methodologies.^7^

By achieving these innovations, scientists retain maximum flexibility to use the system with a variety of tags and at a speed that allows for faster experimentation, making the system ideal for investigating unknown cellular functions and processes and novel drug therapies. To test this application, we created two multiplexed cell lines to study proteins from the severe acute respiratory syndrome coronavirus 2 (SARS-CoV-2).

The recently emerged SARS-CoV-2 is a highly contagious virus that causes the disease COVID-19, a respiratory illness that can induce severe morbidity and mortality in affected patients and for which there are no good therapeutic options available.^13^ The genome of the virus encodes information for 27 structural and non-structural proteins.^14^ These include proteins for viral replication, viral particle formation and assembly, and multiple regulatory proteins. The function of most of these proteins is not fully known.^15^ Therefore, live-cell studies using cells expressing these viral proteins could be advantageous for better understanding their function and/or to identify potential drug targets for developing novel antivirals.

In this work, we compared the effect of seven SARS-CoV-2 viral proteins that interact with vesicle trafficking proteins in human cells^16^ to understand better their role on autophagy, mitochondrial dynamics, and cell growth by using live-cell high-content imaging analysis with two multiplex gene-edited cell lines (without the use of chemical staining or antibodies).

With these cell models, we were able to identify the protein ORF3a from SARS-CoV-2 as a potent inhibitor of autophagy, inducer of mitochondrial relocalization, and a growth inhibitor. This demonstrates the benefits of the FAST-HDR system for developing multiplex cellular models for live-cell studies to understand cells' biological functions by simplifying, accelerating, and enhancing the process for utilizing CRISPR for tagging endogenous genes.

## Methods

### FAST-HDR plasmid vector development

The HDR plasmids were built on a pUC57 backbone plasmid using synthetic DNA fragments (gBlocks; Integrated DNA Technologies, Skokie, IL) and standard molecular biology techniques. The HDR plasmids were maintained in *Escherichia coli* DB3.1 competent cells (Abbexa Ltd., Cambridge, United Kingdom). The functional modules were organized from 5′ to 3′ as follows: first, a CcdB expression cassette flanked by *Kpn*I recognition sites; second, a protein tag sequence flanked by *Eco*RI recognition sites and in frame with a P2A peptide sequence; third, a eukaryotic antibiotic resistance gene flanked by *Xba*I recognition sites; fourth, a MALAT 3′ sequence to provide mRNA stabilization^17^; and finally, a second CcdB expression cassette flanked by *Bam*HI recognition sites.

This design facilitates the rapid modification of the plasmid to include any protein tag or antibiotic resistance gene by digesting the plasmid with *Eco*RI or *Xba*I, respectively and performing Gibson assembly with the desired DNA sequence. The FAST-HDR plasmid vector system is available in the Addgene plasmid repository with the following ID numbers: 167205, 167206, 167207, 167208, 167209, and 167210.

### Cloning of recombination arms into the FAST-HDR system

The recombination arms were obtained as synthetic DNA fragments from Integrated DNA Technologies, and their sequences are provided in Supplementary Table S1. The recombination arms of each gene target were designed by selecting 400–660 nt from the 5′ and 3′ sites around the expected DSB. The left recombination arm (5′ end) was designed to end with the last codon of the target gene without including the stop codon to allow the in-frame expression of the protein tag and the antibiotic resistance gene. The right recombination arm (3′ end) was designed to start at the first nucleotide after the DSB. The recombination arms also included flanking sequences (30 bp) at the 5′ and 3′ ends to enable directional cloning by the Gibson assembly method, and all the recombination arms were compatible with all the plasmids described in this work.

To allow insertion of the recombination arms into the FAST-HDR vector system, 500 ng of the plasmid was digested with *Kpn*I and *Bam*HI in a 20 μL reaction for 1 h using FastDigest enzymes (Thermo Fisher Scientific, Waltham, MA). The recombination arms were re-suspended in TE buffer at 25 ng/μL. Finally, the Gibson assembly reaction was performed for 15 min at 50°C using 2 μL of the digested plasmid, 1.5 μL of each recombination arm, and 5 μL of NEBuilder HiFi DNA Assembly Master Mix (New England Biolabs, Ipswich, MA).

Finally, 3 μL of the Gibson assembly reaction was used to transform 5-alpha chemically competent *E. coli* cells (New England Biolabs). After plasmids extraction, the absence of mutations in the recombination arms was verified by Sanger sequencing by using the following primers: for the left arm, F 5′-GCA GAT TGT ACT GAG AGT GCA CCA TA-3′; for the right arm, F 5′-CAG GTT TTG CTT TTT GGC CTT TCC C-3′.

### CRISPR-Cas9 and single guide RNA expression

The plasmid expressing eSpCas9(1.1)^18^ was a gift from Feng Zhang (Addgene plasmid #71814). The U6 promoter was replaced with a tRNA promoter^19^ to facilitate the expression of single guide RNA (sgRNA). For this purpose, the plasmid was digested with *Psc*I and *Xba*I, and a synthetic DNA construct containing the tRNA sequence plus a CcdB selection cassette flanked by *Bbs*I sites was inserted by Gibson assembly.

This insertion facilitated fast cloning by Gibson assembly of the sequence encoding the sgRNA guides using 80 bp single-stranded DNA ultramer oligos (Integrated DNA Technologies). For this, a 4 nM ultramer oligo was re-suspended in 40 μL of TE buffer to make a stock solution. Then, 1 μL of the stock solution was diluted with 250 μL of Nuclease-free water to make the working ultramer oligo solution.

Finally, the Gibson assembly reaction was performed for 15 min at 50°C using 2 μL of the digested (*Bbs*I) plasmid, 1.5 μL of the diluted oligo, 1.5 μL of Nuclease-free water, and 5 μL of NEBuilder HiFi DNA Assembly Master Mix (New England Biolabs). Finally, 3 μL of the Gibson assembly reaction was used to transform 5-alpha chemically competent *E. coli* cells (New England Biolabs). The 20 bp sgRNA guides for each target were selected with the CRISPR module of the Benchling online platform and are included in Supplementary Table S1.

### Cell culture

HEK293T, HCT116, and HeLa cells were acquired from ATCC (ATCC, Manassas, VA; cat. #CRL-3216, #CCL-247, and #CCL-2, respectively). HEK293T and HeLa cells were grown in Dulbecco's modified Eagle's medium (DMEM) supplemented with 10% fetal bovine serum (FBS), 100 IU/mL penicillin, and 100 μg/mL streptomycin at 37°C and 5% CO_2_. HCT116 cells were grown in McCoy medium supplemented with 10% FBS, 100 IU/mL penicillin, and 100 μg/mL streptomycin at 37°C and 5% CO_2_.

### Cell transfection and antibiotic selection

HEK293T cells (4.5 × 10^5^) were transfected with jetPRIME (Polyplus-transfection; Illkirch, Strasbourg, France) following the manufacturer's protocol. A total of 1 μg of each plasmid (eSpCas9[1.1] and HDR Vector) was used per well of a six-well plate. For transfections that required more than one HDR vector, 700 ng of each plasmid was used. HCT116 or HeLa cells (1 × 10^6^) were electroporated with a Neon electroporation system (Invitrogen, Carlsbad, CA) following the manufacturer's recommendations.

Seventy-two hours after transfection or electroporation, the cells were harvested and seeded on a 100 mm dish containing a selection antibiotic. The culture medium was changed every 48 h, and the selection antibiotics were used at the following concentrations: puromycin at 3 μM, blasticidin at 10 μg/mL, and zeocin at 100 μg/mL.

### Flow cytometry detection of gene-edited cells with fluorescent tags

HEK293T (4.5 × 10^5^) were transfected with plasmid combinations for tagging three endogenous genes (*H3F3B, TUBB,* and *ATP5B*) individually or the three genes at the same time with three fluorescent proteins (mRuby3, mClover3, and mTagBFP2, respectively) as described above. Seventy-two hours post transfection, the cells were washed with Dulbecco's phosphate-buffered saline (DPBS) and harvested with Tryple Express (Thermo Fisher Scientific) for 5 min at 37°C. The cell pellet was collected by centrifugation (2 min at 500 *g*) and re-suspended in FluoroBrite DMEM (Thermo Fisher Scientific) with 10% (v/v) FBS (Corning Corp., Corning, NY).

Similarly, transfected cells that were selected with eukaryotic antibiotics (zeocin, puromycin, and blasticidin) starting at 72 h post transfection and selected after 10 days of treatment were also harvested for analysis. The cells were diluted at 1 × 10^6^ cells in 280 μL of FluoroBrite DMEM with FBS for flow cytometry analysis and were maintained on ice before analysis. Sample data were collected on a BD FACSAria II running BD FACSDiVa software v.7 (BD Biosciences, Franklin Lakes, NJ).

Fluorescent proteins were excited at 488 nm (mClover3), 532 nm (mRuby3), and 405 nm (TAGBFP2) and detected at emission maxima/bandpass values of 525/50 nm, 575/25 nm, and 450/50 nm, respectively. Compensation values were generated using single-color controls for all fluorescent proteins employed and applied to all experimental data profiles. A total of 1 × 10^5^ events were analyzed per sample, and unmodified HEK293T cells were used as control. Autofluorescence baselines (<1 × 10^3^ fluorescent units) for relevant parameters were established with non-modified HEK293T cells. These experiments were repeated three independent times. Data analysis was performed using FlowJo software v10.7.2. (FlowJo LLC, Ashland, OR).

### Validation of the integration of recombinant tags into the genomic DNA and insertion/deletion analysis

Genomic DNA was extracted from modified cell lines using QuickExtract DNA extraction buffer (Epicentre Technologies, Madison, WI) according to the manufacturer's protocol. A set of gene-specific primers were designed for each target using the NCBI primer design tool (https://www.ncbi.nlm.nih.gov/tools/primer-blast/; Supplementary Fig. S1A and Supplementary Table S2). The forward and reverse primers were designed to anneal approximately ∼750 bp upstream and downstream, respectively, of the CRISPR targeting site of each gene under study. Additionally, a universal reverse primer that annealed to the linker of the HDR plasmid was included in every polymerase chain reaction (PCR) mixture to generate a PCR product of ∼750 bp if there was insertion of the recombinant DNA at the targeting site.

The frequency of insertions and deletions (indels) in the untagged alleles after C-terminal tagging (CRISPR + FAST-HDR) and antibiotic selection were determined for two cell lines: HEK293T with Histone 3.3 (*H3F3B*) tagged with mRuby3, and HEK293T with β-tubulin (*TUBB*) tagged with mClover3. In addition, we analyzed unmodified HEK293T cells as a control. For this, the DNA of studied cells was extracted using QuickExtract DNA extraction buffer following the manufacturers' protocol.

A PCR was set up (with Q5 High-Fidelity polymerase from New England BioLabs) to amplify a region containing the sgRNA recognition site sequence for indel frequency detection after CRISPR DSB only in unmodified alleles. The *H3F3B* PCR product (353 bp) was amplified with the following primers: forward 5′-AGG CTA GCG AAG CGT ACC TG-3′; reverse 5′-ATC ACC CAT CCC TTC TGC AT-3′. The *TUBB* PCR product (340 bp) was amplified with the following primers: forward 5′-GGC TGA GAG CAA CAT GAA CG-3′; reverse 5′-CAC CCA GAA TGG CAG AAA CC-3′.

The PCR products were verified by agarose gel analysis to generate a single band and were purified with Wizard^®^ SV Gel and the PCR Clean-up System from Promega (Madison, WI) following the manufacturer's instructions. The PCR products were eluted with molecular biology grade water, quantitated with a Nanoquant™ plate in a Tecan Spark plate reader (Tecan, Männerdorf, Switzerland), and diluted to 20 ng/μL with water. The PCR samples were submitted to GENEWIZ (South Plainfield, NJ) for next-generation sequencing.

The PCR samples were analyzed with a 2 × 250 bp Illumina sequencing reaction without fragmenting the amplicons. The number of amplicon reads per sample was required to be >50,000 to be considered for analysis. The FastQ sequencing files of each PCR reaction were analyzed with the Cris.py^20^ algorithm to determine the frequency of indel events in the PCR amplicons.

### Confocal live-cell microscopy

The Operetta CLS Confocal High Content Imaging System (PerkinElmer, Waltham, MA) was used for single and time-lapse image acquisition. The fluorescent proteins were detected with the following excitation and emission filter combinations: mTagBFP2 (Ex-405, Em-440), mClover3 (Ex-480, Em-513), and mRuby3 (Ex-558, Em-590). The images were acquired with a 20 × , 40 × , or 63 × water immersion objective and 200–500 ms of acquisition time per fluorescent channel. All confocal images in this work were acquired with live cells grown in DMEM without phenol red (Thermo Fisher Scientific) supplemented with 10% FBS on 96- or 384-well CellCarrier Ultra plates (PerkinElmer).

For time-lapse photography of multiplex HEK293T cells treated with staurosporine or paclitaxel, cells were kept under environmentally controlled conditions (37°C and 5% CO_2_), and images were acquired every 10 min. All compounds under study were administered 50 min after the start of image acquisition at the following concentrations: staurosporine, 500 nM; paclitaxel, 20 nM.

### Airyscan super-resolution microscopy

HEK293T cells with nuclear (H3.3-mRuby3), microtubule (β-tubulin-mClover3), and mitochondrial (ATP5B-mTagBFP2) labeling were grown in phenol red-free DMEM supplemented with 10% FBS, 2 mM l-glutamine, 100 IU/mL penicillin, and 100 μg/mL streptomycin at 37°C and 5% CO_2_. For Airyscan imaging, cells were plated onto no. 1.5, 35 mm MatTek imaging chambers (MatTek, Ashland, MA) precoated with 400–600 μg/mL of Matrigel (Corning Corp.), and cells were seeded to achieve ∼60% confluency at the time of imaging.

Cells were imaged in FluoroBrite DMEM (Thermo Fisher Scientific) with 10% (v/v) FBS (Corning Corp.). Airyscan imaging was performed using a Zeiss 880 (Carl Zeiss AG, Oberkochen, Germany) outfitted with an Airyscan module and incubation chamber held at 37°C and 5% CO_2_. Data were collected using a 63 × 1.4 NA objective and immersion oil optimized for 37°C (Carl Zeiss AG). Colors were collected sequentially to minimize crosstalk, and Airyscan processing was performed using the Airyscan module in the commercial ZEN software package (Carl Zeiss AG). A rolling ball background subtraction with a radius of 50 pixels was subsequently applied using Fiji software (National Institute of Health, Bethesda, MD).

### Luciferase live-cell detection

The NRF2-NanoLuc HCT116 cell line was used to evaluate the expression of NanoLuc-tagged NRF2 protein. For this purpose, 1 × 10^4^ cells in 100 μL of culture media were seeded onto 96- or 384-well white plates, and 16 h later, the cells were treated with Enduren™ live-cell luciferase substrate (Promega) at a final concentration of 40 μM. Ninety minutes later, the plates were transferred to a Tecan Spark plate reader (Tecan) with environmental control (37°C and 5% CO_2_) to detect the luciferase signal. The luciferase signal of every well under study was tracked every 15 min with an integration time of 1 s per well, and the experiments were continued for 23 h.

To evaluate the effect of tert-butyl hydroperoxide (TBHP; 25 μM), sulforaphane (SFN, 6.25 μM), and tert-butyl hydroquinone (TBHQ; 25 μM) on NRF2 expression, the cells were treated with the compounds 90 min after the addition of the Enduren™ substrate and immediately transferred to the plate reader to start signal acquisition. Data were acquired with Spark control software and exported to Microsoft Excel or GraphPad Prism v6 (GraphPad Software LLC, La Jolla, CA) for analysis.

### Generation of plasmids for mammalian overexpression of tagged SARS-CoV-2 proteins

The protein sequence of seven SARS-CoV-2 proteins (NSP6, NSP8, N protein, E Protein, M Protein, ORF8, and ORF3a) were obtained from the genomic sequence of the Severe Acute Respiratory Syndrome Coronavirus 2 Wuhan-Hu-1 Isolate (NCBI reference sequence NC_045512.2).

For protein overexpression in mammalian cells, the pTHC plasmid (Promega) was modified to contain a minimal EF1a promoter, a Ccdb prokaryotic selection gene, and a C-term miRFP670 fluorescent protein tag. The genetic constructs to encode each of the seven viral proteins were generated as synthetic DNA fragments (gBlocks from Integrated DNA Technologies) and cloned into the modified plasmid (EC-miRFP670) after digestion of the plasmid with *Eco*RI and the Gibson assembly reaction. All the new genetic constructs were validated by Sanger DNA sequencing.

### Overexpression of SARS-CoV-2 proteins for high-content confocal microscopy

The HeLa cell line with triple endogenous labeling of nucleus (Histone 1-mtagBFP2), microtubules (β-tubulin-mClover3), and the autophagy receptor protein (SQSTM1-mRuby3) as well as the HEK293T cells with labeled nucleus (H3.3-mRuby3), microtubules (β-tubulin-mClover3), and the mitochondria (ATP5B-mTagBFP2) were used for overexpressing seven SARS-CoV-2 fluorescent tagged proteins by transient transfection, with an empty EC-miRFP70 as a control. Briefly, the Jetoptimus transfection reagent (Polyplus-transfection; Illkirch) was used in a reverse transfection reaction with endotoxin-free plasmids as indicated by the manufacturer.

The cells (1 × 10^4^ in 100 μL per well) were seeded in triplicate on 384-well CellCarrier Ultra plates (PerkinElmer) in FluoroBrite DMEM with 10% FBS and Glutamax (Thermo Fisher Scientific). The transfected cells were used for live-cell confocal imaging acquisition with an Operetta CLS Confocal High Content Imaging System (PerkinElmer) with environmental control (37°C and 5% CO_2_). For this, the culture media was exchanged 14 h after transfection, and images were acquired in four independent channels (mTagBFP2 [Ex-405, Em-440], mClover3 [Ex-480, Em-513], mRuby3 [Ex-558, Em-590], and miRFP670 [Ex-642, Em-670]).

For HeLa cells, the images were captured every hour for 24 h by using a 40 × water immersion objective. Four images (with four different channels) were acquired per well with the same coordinates in every well. The experiment was repeated three independent times. The image data analysis was performed using Harmony v4.8 software (PerkinElmer).

The image analysis included the following sequential steps: (1) identification of the cell nucleus (Histone 1-mtagBFP2); (2) identification of the cytoplasm boundaries of identified cell nucleus with TUBB-mClover3; (3) identification of cells overexpressing recombinant proteins (miRFP670); and (4) identification of spots (puncta) in the cytoplasm of cells with or without recombinant protein overexpression for the detection of autophagic vesicles (SQSTM1-mRuby3). For time-lapse imaging of multiplex HeLa cells overexpressing tagged ORF3a or miRFP670 (Supplementary Video V3), a similar procedure was applied, but the images were captured every 20 min for 28 h.

The imaging of triple-edited HEK293T cells overexpressing the seven tagged SARS-CoV-2 proteins was done by time-lapse imaging by using four independent channels (mTagBFP2 [Ex-405, Em-440], mClover3 [Ex-480, Em-513], mRuby3 [Ex-558, Em-590], and miRFP670 [Ex-642, Em-670]). The images were captured 14 h after transfection every 20 min for 24 h. The image analysis routine for identifying cells with or without a large mitochondrial cluster was built with Harmony software with the following steps: first, the cell nuclei were identified; then, the cytoplasm boundaries of each nuclei were identified; and finally, a mitochondria cluster in the cytoplasm >30 μm^2^ was identified. This routine facilitated individual live-cell counting and identification of all the cells with or without a large mitochondrial cluster.

### Western blotting

HeLa cells (2 × 10^5^ cells per well) were transfected on 12-well plates with the plasmids encoding seven miRFP670-tagged SARS-CoV-2 proteins and a control miRFP670 plasmid. For this, we used the Jetoptimus transfection reagent as described above. Additionally, one well with non-transfected cells was treated with hydroxychloroquine (25 μM) for 24 h as an autophagy inhibitor control. Forty-eight hours after transfection, the cells were washed twice with cold PBS and harvested with a plastic cell lifter in 1 mL of cold PBS and centrifugation at 800 *g* for 2 min.

The cell pellet was lysed with M-PER lysis buffer containing protease inhibitors (Thermo Fisher Scientific) for 20 min on ice, followed by vortexing and centrifugation for 25 min at 20,000 *g* at 4°C. The lysate supernatant protein concentration was quantitated with the Bradford assay (Bio-Rad Laboratories, Hercules, CA). Protein samples were separated for detecting LC3B by using the Simple Western WES™ system from ProteinSimple (Biotechne, Minneapolis, MN) following the manufacturer's recommendations. Rabbit monoclonal antibodies for LC3B (cat. #12741) and B-actin (cat. #4970) were obtained from Cell Signaling Technologies (Danvers, MA).

### Reproducibility and statistical analysis

All figures in this work are representative of at least three independent experiments. Differences between control and treated samples were performed by using two-way analysis of variance within GraphPad Prism v8 (GraphPad Software, La Jolla, CA).

## Results

### Construction of a plasmid backbone system for the fast assembly of donor templates

**FIG. 1.**
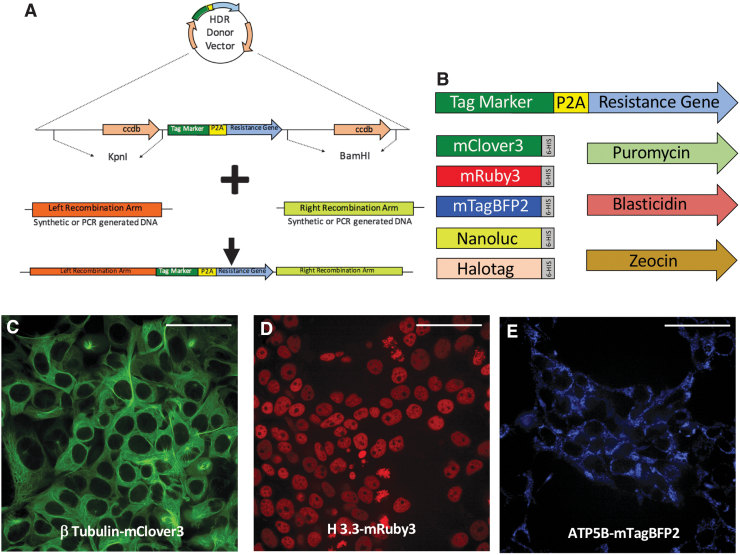
The FAST-HDR vector system. **(A)** Diagram of the general components of the plasmid that facilitates the rapid construction of donor templates for homologous recombination. The plasmid is first digested with restriction enzymes to cut the segments with CddB cassettes (in this case, *Kpn*I and *Bam*HI). Previously designed synthetic recombination arms are cloned into the digested backbone plasmid using the Gibson assembly method. **(B)** Diagram of the protein tags and selection antibiotics that are included in the FAST-HDR system. **(C)**–**(E)** HEK293T cell lines with labeling of **(C)** β-tubulin with mClover3, **(D)** H3.3 with mRuby3, and **(E)** ATP5B with mTagBFP2. Bar = 50 mm.

The FAST-HDR system was constructed by combining three existing technologies to accelerate gene cloning. First, double cloning of the 5′ and 3′ recombination arms was performed in a single step by promoting the replacement of two copies of a lethal gene for *E. coli*, *ccdB*,^21^ thus allowing only the growth of colonies with the correct insertions of the arms. Second, the 5′ and 3′ recombination arms were provided as sequence-verified synthetic dsDNA. Third, the Gibson assembly method^22^ facilitated the correct insertion of the 5′ and 3′ recombination arms in a single reaction (Fig. 1A). Thus, this streamlined method reduces the time for generating an HDR plasmid from ∼3 weeks to only 1 day and requires <30 min of hands-on time.

In addition, the system is now set up for allowing the insertion of multiple protein tags for downstream applications, such as fluorescence microscopy, luminescence, and protein purification (Fig. 1B). The current system is preconfigured with five different protein tags and three eukaryotic antibiotics. The system can also be easily modified to include any protein tag or eukaryotic resistance gene that a researcher might need.

### Fast generation of cell lines with tagged endogenous genes

We tested the FAST-HDR plasmids by generating multiple cell lines that highlight the benefits of this system and present a broad spectrum of applications. First, we created three plasmid backbones to allow the tagging of endogenous genes with red fluorescent protein mRuby3,^23^ the green fluorescent protein mClover3,^23^ and the blue fluorescent protein mTagBFP2.^24^ Each backbone plasmid was also built to contain a different eukaryotic resistance gene (zeocin, puromycin, and blasticidin, respectively).

Next, we targeted *HEK293T* cells with CRISPR-Cas9 and the HDR plasmids to modify endogenous genes encoding proteins with well-defined subcellular localizations. In particular, we C-terminally tagged the genes encoding Histone 3.3 (H3.3, nucleus), β-tubulin (microtubules), and ATP synthase subunit β (ATP5B; mitochondria), with mRuby3, mClover3, and mTagBFP2, respectively (Fig. 1C–E and Supplementary Fig. S1). In all cases, the new HDR plasmids and workflow (Supplementary Fig. S2) allowed the selection of modified cell lines between 9 and 14 days after assembling the HDR plasmids.

We determined the efficiency of the gene tagging process 72 h post transfection and before antibiotic selection by detecting fluorescent cells via flow cytometry. We found an editing frequency between 21% and 44%: H3.3-mRuby3 (40 ± 3.9%), β-tubulin (42 ± 9.6%), ATP5B (26 ± 4.8%; Supplementary Fig. S3). We repeated the flow cytometry assay for H3.3-mRuby3 and β-tubulin-mClover3 after antibiotic selection to show the specificity of the selection, and we found >98% of the cells in the population with fluorescent signals (Supplementary Fig. S4).

We also analyzed the presence of indels due to CRISPR DSB in the untagged alleles of two edited HEK293T cell lines (Histone 3.3-mRuby3 and β-tubulin-mClover3) after antibiotic selection. We found that the presence of indels (92% for *H3F3B* and 79% for *TUBB*) was common in the untagged alleles.

### Multiplexing insertions of three labeling tags with the FAST-HDR system

To demonstrate the applicability of multiplexing with the FAST-HDR system, we generated two HEK293T cell lines each with three tagged endogenous genes with different fluorescent reporters. The multiplex gene tagging efficiency of three targets was found to be ∼3% before antibiotic selection and >94% after selection by using flow cytometry (Supplementary Fig. S5). Furthermore, because these cell lines already have endogenous proteins labeled with fluorescent tags, they do not require the use of immunofluorescence or fluorescent staining to generate images for high-content imaging analysis. Thus, the system eliminates the additional time, cost, and effort required to detect the proteins of interest used in existing processes and allows evaluation of cellular changes at different time points without the requirement for fixation.

**FIG. 2.**
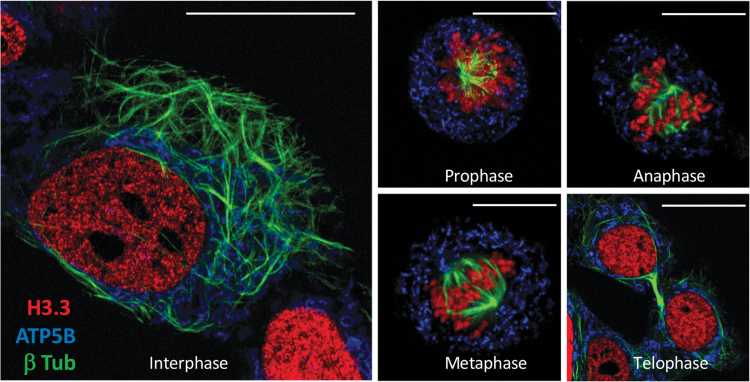
The FAST-HDR system facilitates the development of cell lines with multiple endogenously labeled genes. Evaluation of the mitotic states of HEK293T cells with nuclear (H3.3-mRuby3), microtubule (β-tubulin-mClover3), and mitochondrial (ATP5B-mTagBFP2) labeling by super-resolution confocal microscopy. Bar = 15 mm.

**FIG. 3.**
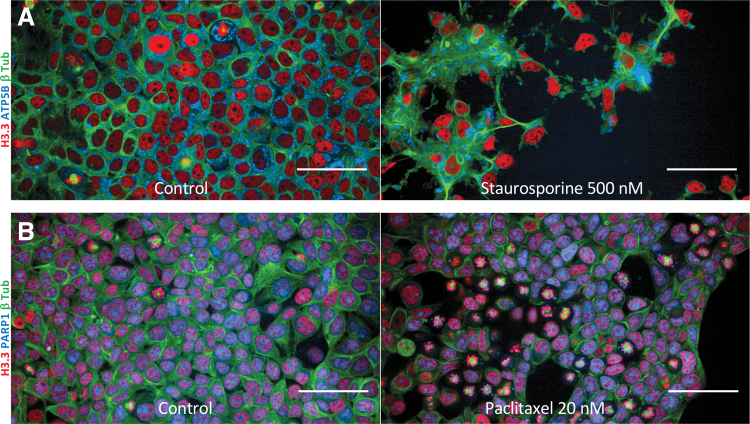
Use of multiplex cell lines for time-lapse confocal imaging without antibodies or chemical staining. **(A)** The growth of HEK293T cells with nuclear (H3.3-mRuby3), microtubule (β-tubulin-mClover3), and mitochondrial (ATP5B-mTagBFP2) was evaluated by time-lapse confocal microscopy for 13 h with and without an apoptosis inducer (staurosporine). The complete time-lapse sequence can be seen in Supplementary Video V1. **(B)** The growth of an HEK293T cell line with endogenous labeling of PARP1-mTagBFP2, H3.3-mRuby3, and β-tubulin-mClover3 was evaluated by time-lapse confocal microscopy for 13 h with and without a microtubule-stabilizing drug (paclitaxel). The complete time-lapse sequence can be seen in Supplementary Video V2. Bar = 50 mm.

Such cell lines, with multiple fluorescent tags, are an excellent tool for super-resolution microscopy (Fig. 2) or live-cell confocal microscopy (Fig. 3). In our case, we evaluated the growth of these cell lines by time-lapse microscopy (13 h) after treatment with staurosporine, a well-known inducer of apoptosis (Fig. 3A and Supplementary Video V1). We then visualized the induction of cytoplasmic fragmentation and the transition into apoptotic bodies. We also studied the effects of paclitaxel, a microtubule-stabilizing drug, and found that cells treated with paclitaxel going through mitosis cannot execute the final two-daughter cell separation step (cytokinesis) as occurs with untreated control cells (Fig. 3B and Supplementary Video V2).

### High-content imaging analysis of multiplexed gene-tagged cell lines overexpressing SARS-CoV-2 recombinant proteins

To validate the applicability of multiplex gene-tagged cell lines for high-content imaging analysis further, we created a HeLa cell line for evaluating the formation and accumulation of autophagic vesicles in individual cells. Autophagy is a physiological process that regulates the formation of intracellular vesicles containing cellular components that require degradation.^25,26^ Autophagy facilitates the controlled degradation of damaged cellular organelles, the recycling of cellular components during starvation, and the degradation of intracellular pathogens. Thus, processes that affect autophagy can induce pathological consequences.^27^

In the HeLa cell line, we tagged a nuclear protein (Histone 1-mtagBFP2), a cytoskeleton marker (β-tubulin-mClover3), and an autophagy receptor protein (p62/SQSTM1- mRuby3). It is known that viral pathogens can alter physiological autophagy by inhibiting the process to prevent the degradation of endogenous viral particles in formation,^28–31^ or they can induce autophagy as a pro-survival mechanism.^32–35^

We selected seven SARS-CoV-2 proteins (NSP6, NSP8, ORF8, N protein, M protein, E protein, and ORF3a) that interact with vesicle trafficking proteins in human cells^16^ for recombinant overexpression in the triple gene-edited HeLa cell line to study autophagic vesicle formation in live cells. For detecting overexpressed viral proteins in these HeLa cells, we added a c-terminal miRFP670 fluorescent protein to all the recombinant proteins (Supplementary Fig. S6).

This approach allowed us to use a total of four independent fluorescent channels (cell nucleus [H1-mtagBFP2], cytoskeleton [TUBB-mClover3], autophagic vesicles [p62-mRuby3], and recombinant viral proteins [miRFP670]) for image acquisition, cellular segmentation, and differential analysis of autophagic vesicles in individual cells overexpressing the recombinant viral proteins during a period of 24 h.

**FIG. 4.**
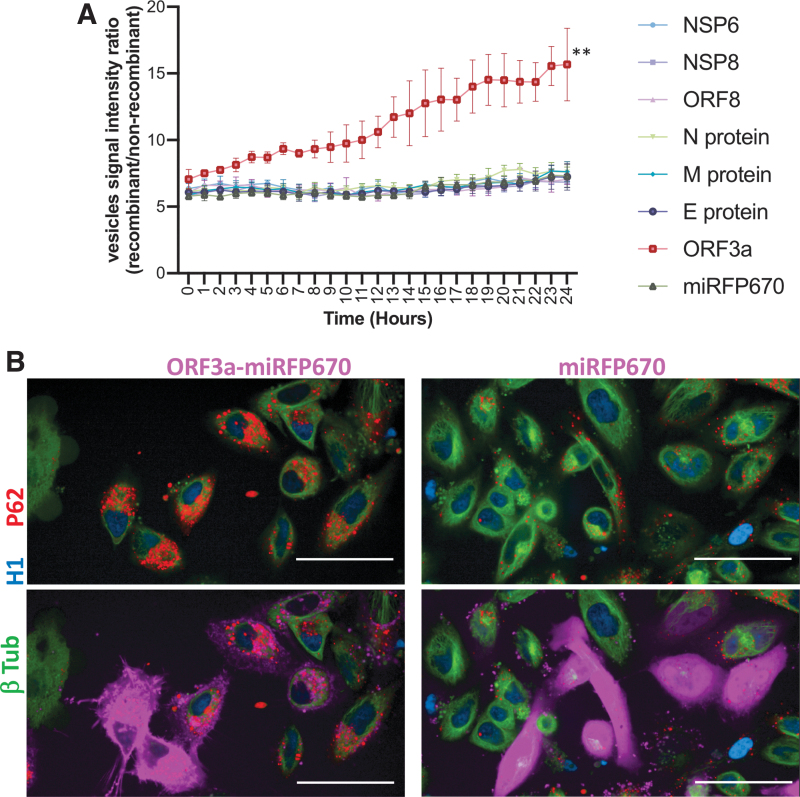
The expression of severe acute respiratory syndrome coronavirus 2 (SARS-CoV-2) ORF3a promoted a time-dependent accumulation of autophagic vesicles. **(A)** Seven SARS-CoV-2 proteins with a c-terminal miRFP670 and miRFP670 alone as a control were overexpressed by transient transfection into a HeLa cell line containing endogenous tagging of β-tubulin (mClover3), Histone 1 (mtagBFP2), and P62/SQSTM1 (mRuby3). Fourteen hours after transfection, the signal intensity of autophagic vesicles (P62/SQSTM1-mRuby3) of individual cells overexpressing each recombinant protein was captured every hour for 24 h with automated high-content confocal microscopy. The signal intensity of the autophagic vesicles from cells overexpressing the recombinant proteins was normalized against the intensity of autophagic vesicles in cells not overexpressing the proteins in the same image. Differences were analyzed by two-way analysis of variance (ANOVA), and *p* < 0.05 was considered statistically significant. **p < 0.001; error bars represent ±standard deviation (*SD*) of triplicate wells. These data are representative of three independent experiments. **(B)** Confocal image of multiplex gene-tagged HeLa live cells overexpressing SARS-CoV-2 ORF3a-miRFP670 or miRFP670 alone as control for detecting autophagic vesicles (P62/SQSTM1-mRuby3) 48 h after transient transfection. Bar = 50 mm.

We were able to perform image analysis of individual cells (3,456 images per recombinant protein) for tracking autophagic vesicles' signal intensity in cells overexpressing each of the seven SARS-CoV-2 proteins. Each of these were compared to the control that expressed miRFP670 alone, as well as with cells that were not actively overexpressing the recombinant proteins—all in the same image. We found that the overexpression of SARS-CoV-2 ORF3a protein induced a strong time-dependent increase in the signal intensity of autophagic vesicles when compared against all the other proteins (Fig. 4A and B and Supplementary Video V3).

**FIG. 5.**
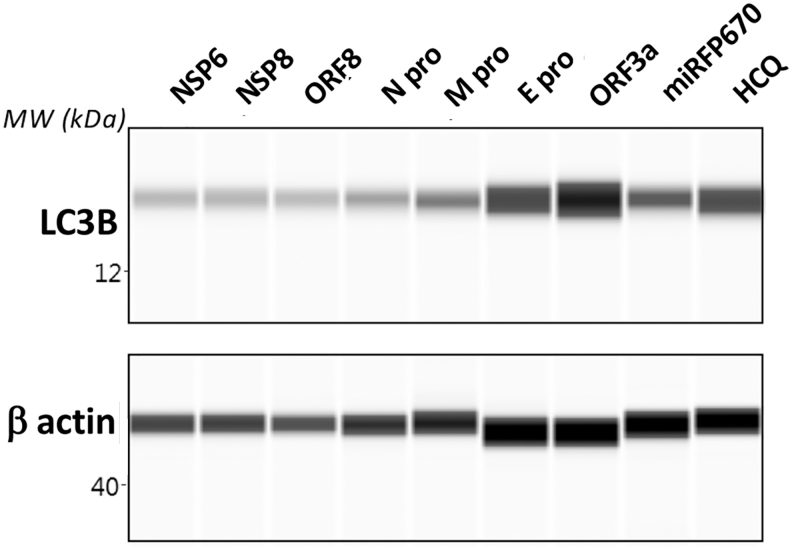
The expression of SARS-CoV-2 ORF3a inhibited autophagy. Western blot for LC3B from HeLa cells overexpressing seven SARS-CoV-2 miRFP670-tagged proteins, and miRFP670 alone (control) 48 h after transient transfection. Hydroxychloroquine (HCQ) treatment of untransfected cells was used as a positive control for inhibiting autophagy. The image is representative of three independent experiments.

To determine if the increased intensity in signal from autophagic vesicles in cells overexpressing ORF3a occurred due to inhibition or activation of autophagy, we performed Western blotting for detecting the concentration of the protein LC3B in transfected cells with all seven SARS-CoV-2 proteins and included cells treated with an autophagy inhibitor as a positive control. LC3B is a component of autophagic vesicles that is degraded during autophagy.^36^ We found that cells overexpressing ORF3a contain high accumulation of LC3B when compared to cells overexpressing all the other proteins (Fig. 5), thus indicating strong inhibition of autophagy similar to what was found in cells treated with an autophagy inhibitor.

It has been reported that during viral infections, cellular organelles in the infected cells might suffer structural reorganization to facilitate the process of viral replication.^37,38^ Particularly, mitochondrial dynamics can be altered by certain viruses.^39,40^ We explored the capacity of the seven SARS-CoV-2 proteins described above to alter mitochondrial dynamics, since it had been reported that some of these proteins interact with mitochondrial proteins in human cells.^16^ For this, we overexpressed the seven SARS-CoV-2 proteins in a HEK293T cell line with triple gene tagging (H3F3B-mRuby3-nucleus, TUBB-mClover3-cytoskeleton, ATP5B-mtagBFP2-mitochondria) for evaluating the mitochondrial dynamics in live cells and total cell growth over time.

**FIG. 6.**
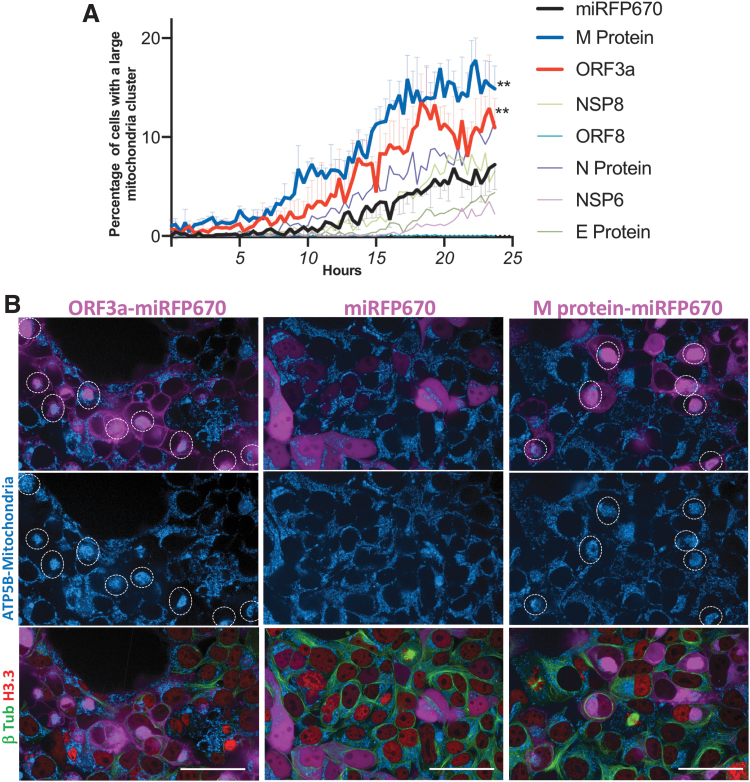
SARS-CoV-2 M protein and ORF3a induced mitochondrial perinuclear clustering. **(A)** Seven SARS-coV-2 miRFP670-tagged proteins and miRFP670 alone as control were overexpressed by transient transfection into a HEK293T cell line containing endogenous tagging of microtubules (β-tubulin-mClover3), nucleus (Histone 3.3-mRuby3), and mitochondria (ATP5B-mtagBFP2). Fourteen hours after transfection, the cells were imaged (four channels) by automated high-content confocal microscopy every 20 min for 24 h. The cells with or without a large mitochondrial cluster in the cytoplasm were detected in every image of the time lapse. Differences were analyzed by two-way ANOVA, and *p* < 0.05 was considered statistically significant. **p < 0.001; error bars (only displayed for M, ORF3a, and miRFP670 to facilitate visualization) represent ±*SD* of triplicate wells. These data are representative of three independent experiments. **(B)** SARS-coV-2 miRFP670-tagged ORF3a, M protein, and miRFP670 alone as control were overexpressed by transient transfection into a HEK293T cell line containing endogenous tagging of microtubules (β-tubulin-mClover3), nucleus (Histone 3.3-mRuby3), and mitochondria (ATP5B-mtagBFP2). Forty-eight hours after transfection, the cells were imaged by live-cell high-content confocal microscopy. White circles highlight mitochondrial clustering in cells overexpressing the recombinant proteins. This image is representative of three independent experiments. Bar = 50 mm.

We overexpressed the seven SARS-CoV-2 proteins by transient transfection, and 14 h after transfection, we started to acquire images every 20 min for 24 h. We created an image analysis routine that detected the formation of large clusters of mitochondria in individual live cells. We were able to find that the overexpression of protein M and ORF3a promotes the relocation of the mitochondria into a single perinuclear cluster in the cytoplasm in cells overexpressing these proteins. By contrast, the mitochondria in cells overexpressing the other proteins or miRFP670 alone as a control had a widely spread cytoplasmic distribution (Fig. 6A and B).

**FIG. 7.**
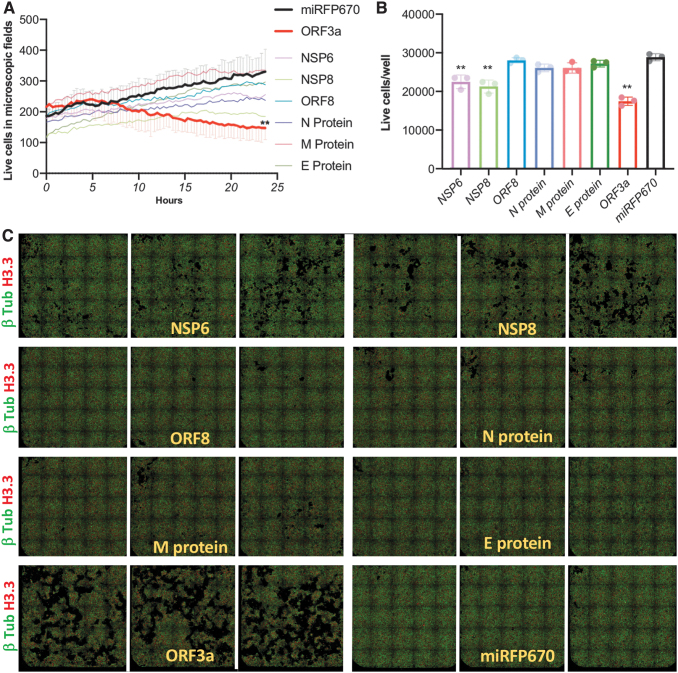
SARS-coV-2 ORF3a protein expression reduced cell growth. Seven SARS-coV-2 miRFP670-tagged proteins and miRFP670 alone as control were overexpressed by transient transfection into a HEK293T cell line containing endogenous tagging of microtubules (β-tubulin-mClover3), nucleus (Histone 3.3-mRuby3), and mitochondria (ATP5B-mtagBFP2). **(A)** Fourteen hours after transfection, the number of live cells in microscopic fields were detected by automated high-content imaging. Live cells were counted by detecting cells with well-defined nucleus and cytoplasm every 20 min for 24 h. Error bars (only displayed for ORF3a and miRFP670 to facilitate visualization) represent ±*SD* of triplicate wells. **(B)** Seventy-two hours after transfection, the total surface of each well (triplicates on a 384-well plate) was imaged with tiled images with high-content confocal microscopy. **(C)** Detection and quantitation of live cells in **(B)**. Differences were analyzed by two-way ANOVA, and *p* < 0.05 was considered statistically significant. **p < 0.0001; error bars represent ±*SD* of triplicate wells. The figure is representative of three independent experiments.

The continuous detection of live cells overexpressing the seven SARS-CoV-2 proteins in a 24-h period allowed us to generate cell growth curves to detect potential changes induced by each of the recombinant proteins. Our analysis indicated that cells overexpressing ORF3a had reduced cell growth rate (Fig. 7A). The time-lapse visual inspection does not suggest the induction of apoptosis by ORF3a overexpression and instead indicates induction of a slower mitotic rate (Supplementary video V4). To confirm these findings, we detected all the live cells in the total area of the wells (384-well plate) at 72 h after transfection and found that the overexpression of ORF3a reduced the cell growth by 39.5% (Fig. 7B and C).

### The FAST-HDR vector system facilitates generating homozygous tagging of target genes

To test whether the FAST-HDR system can be used to generate homozygous labeled cell lines, we cloned the recombination arms into two backbone plasmids with different resistance genes for dual antibiotic selection. We targeted near-diploid HCT116 cells^41^ with a combination of CRISPR-Cas9 and two HDR donor plasmids to tag the transcriptional regulator of the antioxidant response, NRF2 (nuclear factor (erythroid-derived 2)-like 2), with NanoLuc luciferase^42^ at the C-terminus. NRF2 is a low-abundance protein that is tightly regulated by oxidative stress.^43^ We confirmed the tagging of both alleles of *NFE2L2* (Supplementary Fig. 1D).

**FIG. 8.**
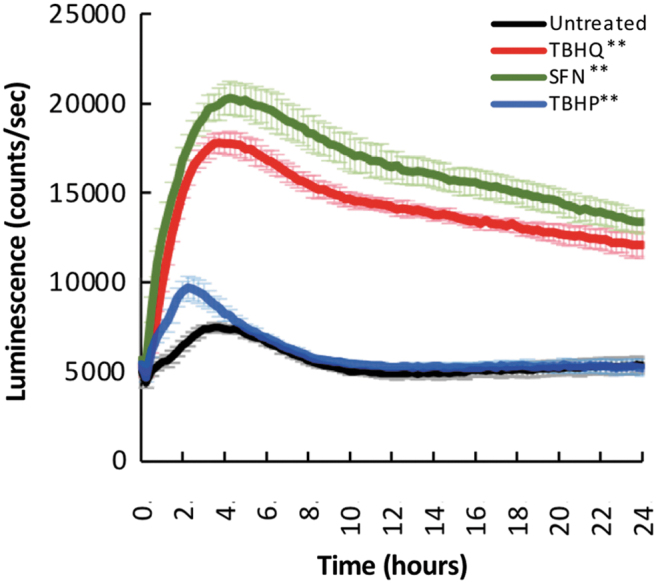
Generation of a cell line with knock-in endogenous labeling of Nrf2 with Nanoluc luciferase. Time-course luminescence produced by an HCT116 cell line with homozygous tagging of the NRF2 protein with NanoLuc, in the presence or absence of tert-butyl hydroquinone (TBHQ), sulforaphane (SFN), and tert-butyl hydroperoxide (TBHP). Data shown are the average and *SD* of six independent experiments. Differences were analyzed by two-way ANOVA, and *p* < 0.05 was considered statistically significant. **p < 0.0001.

This cell line facilitated detection of NRF2 levels in real time in response to an inducer of oxidative stress, TBHP,^44^ or to two compounds that prevent the rapid degradation of NRF2, SFN,^45^ and TBHQ^46^ (Fig. 8). Therefore, the FAST-HDR system can be used to tag proteins of interest homozygously to study their behavior under different conditions.

### The FAST-HDR vector system allows the use of any labeling tag for a target gene

Finally, the system allows for swappable tagging. That is, the single design of recombination arms can be inserted in any of the plasmids within the system to facilitate targeting the same gene with different tags. We confirmed that by cloning one set of recombination arms into several HDR plasmids, the FAST-HDR system can be used to generate multiple cell lines with various modifications of the same target gene.

**FIG. 9.**
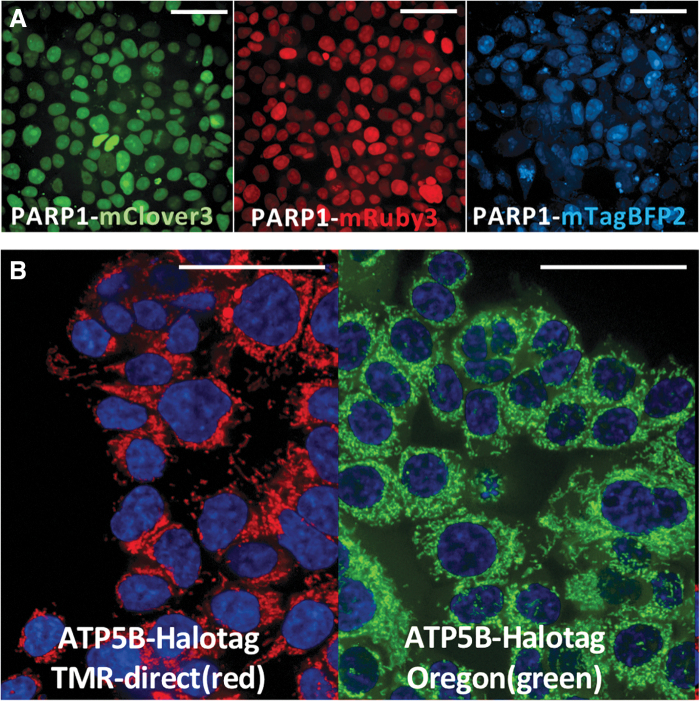
Using the FAST-HDR system to target an endogenous gene with multiple tags. **(A)** Three cell lines (HEK293T) with the *PARP1* gene labeled with different fluorescent tags. **(B)** An HCT116 cell line with homozygous tagging of the ATP5B protein with the HaloTag. For protein visualization, the cells were treated with the HaloTag ligand TMR-direct (red) or Oregon Green and counterstained with Hoechst 33342 to detect the nucleus.

We generated multiple HEK293T cell lines with the endogenous labeling of poly(ADP-ribose) polymerase 1 (PARP1) with three different fluorescent proteins (Fig. 9A). We also generated an HCT116 cell line with homozygous tagging of ATP5B with the purification/labeling tag HaloTag^47^ (Fig. 9B). For this purpose, we used the same recombination arm constructs as for the labeling of ATP5B with mTagBFP2 in Figure 1E. Therefore, the FAST-HDR system offers the flexibility to label the same protein target with different tags, according to the application of the cell line.

## Discussion

In this study, we developed a new donor backbone plasmid system (FAST-HDR) to leverage CRISPR-Cas9 for developing genetically modified cell lines, specifically for inserting labeling tags into multiple genes of interest to track and study their proteins in live cells. We tested the applicability of these cell lines by overexpressing seven SARS-CoV-2 proteins to understand their effect better on autophagy, mitochondrial dynamics, and cell growth as a robust alternative to traditional methods. We found several advantages in using the FAST-HDR system over traditional HDR plasmids for creating gene tagged cell lines.

(1) Each backbone plasmid with a specific labeling tag allowed the incorporation of both the 5′ and 3′ recombination arms in a single enzymatic reaction, thus avoiding the traditional multi-step assembly process.^7^ This feature also increased the speed of obtaining a functional HDR donor template from up to 3 weeks with other methodologies^7^ to only 1 day with FAST-HDR.

(2) The FAST-HDR vector system allowed the in-frame expression of any eukaryotic antibiotic resistance gene for rapid selection of highly rich (>94%) pools of modified cells. The selection of pure cell clones after gene editing typically requires the use of equipment for cell sorting,^9^ which due to its high expense is not available in every laboratory. Alternatively, the isolation of modified clones can be performed by cell selection with resistance cassettes against eukaryotic antibiotics, followed by single-cell dilution. However, this process with existing methods would take several weeks.^7,9^

The choice to work with either cell pools or cell clones will depend on the particular application of the cell line. For cellular assays where the user requires a homogeneous population of homozygous or heterozygous cells, it is recommended that cell sorting or single-cell dilutions be performed to identify clones with the desired characteristics.

For high-content imaging experiments where the image analysis facilitates studying individual cells, users can work with pools of modified cells. This was exemplified recently with an alternative methodology to generate a library of ∼1,300 pool modified cell lines with CRISPR-mediated C-terminal tagging followed by automated image acquisition and image analysis.^48^ However, this alternative approach relies heavily on the use of flow cytometry cell sorting to enrich the population of positively tagged cells.

(3) The system successfully facilitated labeling of up to three target genes in a single cell line (multiplexing), thus allowing the creation of rich cellular models for high-content imaging that did not require the use of staining or immunofluorescence procedures. The use of these triple edited cell lines for microscopy imaging was highly advantageous over conventional cell models because they allowed for the study of endogenous targets expressed under physiological conditions. Furthermore, the system reduced the overall workload by eliminating the time, effort, and expense of using staining or immunofluorescence.

Live-cell analysis allowed the detection of changes in multiple targets over time and integrated well with current high-content imaging devices for multichannel detection and image software analysis.

The FAST-HDR system also facilitates developing donor vectors to modify each allele of a target gene by using two different antibiotic selection genes, as shown in Figure 8. This suggests that creating cellular models where each allele of a target gene can be labelled with a different reporter tag is possible. This allows for the use of complementary reporters tagging the same gene to study multiple characteristics simultaneously. For example, the user can measure the global expression of the target protein with a luminescence assay while also detecting changes in the target protein localization via confocal imaging.

(4) The cassettes for the labeling tag and the mammalian selection marker were designed to be replaceable, thus allowing the easy generation of additional backbones for labeling genes with any type of protein tag and for selecting cell lines with multiple targeted genes (multiplexing). In the current version, we have included three fluorescent tags (mClover3, mRuby3, and mtagBFP2), one luminescent tag (nanoluc), and one purification tag (halotag), as well as three mammalian selection antibiotics cassettes (puromycin, blasticidin, and zeocin). More importantly, we have included clear guidelines for the modification of the system by other researchers interested in specific labeling tags or selection antibiotics.

We conducted live-cell studies with multiplex gene tagged cell lines to study seven SARS-CoV-2 proteins to evaluate their capacity to alter autophagy, mitochondrial dynamics, and cell growth. We used multiplex gene tagged HeLa and HEK293T cells for these studies because the molecular biology of multiple coronavirus proteins has been studied on these cell lines.^49–51^ We selected these seven proteins due to a recent report^16^ that indicated their interaction with human proteins that are part of the vesicle trafficking network or the mitochondria. Since the functions and processes of these SARS-CoV-2 proteins are not fully known, live-cell studies were useful to determine their capacity to alter physiological processes in cellular organelles that change over time.

Of the seven proteins studied, only one protein, ORF3a, had an effect on all three processes. We found that the overexpression of the protein ORF3a inhibits autophagy. In addition, we found that ORF3a also altered mitochondrial dynamics by promoting the relocation of mitochondria into a large perinuclear cluster instead of being widely distributed in the cytoplasm. Lastly, we found that over time (72 h), ORF3a reduced cell growth by ∼40%.

ORF3a (also known as 3a protein) is a protein that is also encoded by the severe acute respiratory syndrome coronavirus 1 (SARS-CoV-1) genome,^52^ which has a 73% similarity with the homologue protein in SARS-CoV-2. A BLASTN search at the NCBI nucleotide database indicates that ORF3a is not present in the genome of the other coronaviruses that cause diseases in humans (alpha coronavirus 229E, NL63 alpha coronavirus, OC43 beta coronavirus, HKU1 beta coronavirus, or Middle East Respiratory Syndrome coronavirus).

In SARS-CoV-1, it has been shown that ORF3a is a protein with multiple biological functions for the downregulation of intracellular innate immunity by promoting the ubiquitination and degradation of Interferon alpha receptor subunit 1 (IFNAR1)^53^ and the inhibition of phosphorylation and translocation of STAT3.^54^

The identification of ORF3a in SARS-CoV-2 in this work as a potent autophagy inhibitor indicates an additional capability for this protein to inhibit a biological process that it is well known for defending cells from viral infections.^55,56^ Two recent reports published post the completion of this work independently confirmed that ORF3a from SARS-CoV-2 is an autophagy inhibitor.^57,58^ ORF3a can inhibit autophagy by affecting the functionality of the homotypic fusion and protein sorting (HOPS) tethering complex by interacting with one of the proteins of the complex called VPS39.^57,58^

The HOPS tethering complex is known to participate in the autophagy pathway by mediating the fusion between autophagosomes and lysosomes. This fusion event is essential for physiological autophagy to continue, as knockdown with siRNA of individual members of the HOPS complex, including VPS39, strongly inhibits autophagy.^59^ A previous report about the interaction of proteins in the SARS-CoV-2 genome with human proteins^16^ supported these findings by showing that ORF3a interacts with VPS11 and VPS39—two proteins from the HOPS tethering complex.

Additionally, Zhang *et al*.^58^ identified that the transmembrane domain of ORF3a is essential for the subcellular localization of ORF3a into lysosomes and also that a sorting motif (YXXΦ) located in the C-terminal portion of ORF3a is required for the interaction with VPS39. The removal of the transmembrane domain or a single amino acid mutation (Y160A) in the YXXΦ motif abolishes the capacity of ORF3a to inhibit autophagy.

The mitochondria play a central role for regulating intracellular antiviral responses via the mitochondria-associated antiviral signaling protein (MAVS). Upon identification of viral RNA by pattern recognition receptor proteins such as RIG-I, the MAVS protein is activated to promote the expression of genes of the interferon I pathway to mount an intracellular antiviral response.^60,61^

The capability of M protein and ORF3a from SARS-CoV-2 to alter mitochondrial dynamics by promoting the formation of a large cluster of the organelle suggests an additional property of these proteins to promote evasion of an intracellular antiviral response, as has been described for other RNA viruses. For example, it is known that there are specific viral proteins that alter mitochondrial dynamics by altering mitochondrial fusion or fission for inhibiting innate immune functions of the mitochondria.^39,62^

A recent report showed that SARS-CoV-2 M protein interacts with RIG-I, MAVS, and TBK1 in HeLa and HEK293T cells, thus inhibiting the activation of the intracellular interferon pathway and facilitating viral replication.^63^ The same report indicates that the membrane domain of M protein is essential for the interaction with RIG-I, but it was not possible to define a single domain that was crucial for the interaction with MAVS, as all the M protein fragments evaluated were able to interact with MAVS. We recommend that future work could elucidate if ORF3a from SARS-CoV-2 alters MAVS signaling and could also aim to identify the host proteins that are affected by M protein and ORF3a to change mitochondrial dynamics.

The current version of the FAST-HDR system has certain limitations. This version can only be used for C-terminal tagging, and the system requires the active expression of the target protein in the cell of interest to facilitate antibiotic selection. The generation of a plasmid template that includes a promoter cassette for a eukaryotic resistance gene or reordering the genetic elements to facilitate N-terminal tagging would likely provide a method to overcome these limitations in the future.

Additionally, the use of CRISPR-Cas9 for genome editing might cause off-target or on-target damage to the DNA of some cells in the treated population.^64^ We try to minimize the possibility of getting off-target effects by carefully selecting unique regions in the targeted genes for inducing CRISPR DSBs and by using an engineered version of CRISPR^18^ with lower off-target potential. Alternatively, the use of CRISPR nickases can further reduce the possibility of damaging unintended sites by off-target effects.^64^

A recent report indicates that CRISPR-Cas9 can also generate unintended on-target DNA damage that can cause micronuclei formation and chromothripsis in a small percentage of treated cells.^65^ This occurs when the CRISPR cut is not repaired, and a portion of a split chromosome remains in isolation, thus promoting the formation of a micronuclei around it. This on-target unintended effect of using CRISPR requires further exploration for the use of CRISPR in clinical applications.

The work of Leibowits *et al*.^65^ did not explore the rate of micronuclei formation or chromothripsis in cells receiving a donor repair template or in cells selected with a eukaryotic antibiotic after incorporating a repair template. Thus, it is hard to anticipate the potential limitation of creating multiplex gene-tagged cellular models with CRISPR and the FAST-HDR system due to the risk of inducing this type of on-target damage. We speculate that due to the low frequency of this on-target damaging effects of CRISPR, the development of cellular models for research with multiplex tagging will not contain a large proportion of cells with large genomic aberrations.

We also detected that on-target indels on the untagged alleles at the intended CRISPR-cutting site were common. This could be due to the higher efficiency of the non-homologous end joining (NHEJ) DNA repair process when compared against the less common HDR repair mechanism.^66^ Thus, the presence of indels in the untagged alleles might affect the function of the encoded protein if the amino acids located at the end of the C-term region are required for the function of the intended protein. If necessary, indels can be limited with the use of methods to inhibit the NHEJ repair mechanism.^66^ We recommend characterizing the functionality of the cellular models for the intended purposes and always validating the discoveries made with gene-edited cellular models with other unmodified cell types.

Lastly, the FAST-HDR system was only tested with seven endogenous genes, but there are currently no limitations that would prevent using the system for high-throughput gene tagging. In fact, recent methodologies that facilitate high-throughput gene tagging limit their use to a specific cell line and only allow targeting one gene with green fluorescent protein.^48,67^ In addition, while the current version of the FAST-HDR system was developed for targeting three genes, the system allows the user to expand to more tagged genes by using additional antibiotic resistance genes with different mechanisms of action that do not overlap with those for puromycin, blasticidin, or zeocin.

## Conclusion

The FAST-HDR system accelerates the construction of gene-tagged cell lines for multiple applications, such as confocal and super-resolution microscopy, the development of reporter cell lines for drug screening, and high-content imaging studies without the use of antibodies or exogenous fluorescent markers. We believe the FAST-HDR system is therefore a versatile methodology for developing endogenous tagging with CRISPR according to the needs of the researcher, as it works on any cell line, facilitates multiplexing, and allows the use of any protein tag.

## Supplementary Material

Supplemental data

Supplemental data

Supplemental data

Supplemental data

Supplemental data

Supplemental data

Supplemental data

Supplemental data

Supplemental data

Supplemental data

Supplemental data

Supplemental data
