# Supplementary material for: Multiplex Gene Tagging with CRISPR-Cas9 for Live-Cell Microscopy and Application to Study the Role of SARS-CoV-2 Proteins in Autophagy, Mitochondrial Dynamics, and Cell Growth
Source: CRISPR J. 2021 Dec 16;4(6):854–71. doi: 10.1089/crispr.2021.0041 (PMC8742308; doi:10.1089/crispr.2021.0041)
Supplement: Supplemental data [file Suppl_TableS1.pdf]

Supplementary Table 1. Sequences of the Synthetic DNA constructs for developing homologous recombination vectors for the genes targeted in this work. The Flanking sequences for Gibson assembly are colored in red.

Gene Target	5’ Arm	3’ Arm	SgRNA sequence (underlined)
ATP5B (ATP synthase subunit B)	GACGTTGTAAAACGACGGCCA<br>GTGGGTACCCGCCACCACGCC<br>TGGCTAATTTTTGTATTTTTAGT<br>AGCCATGGGGTTTCACCATGTT<br>GGCTGGGCTTGTCTCGAACTC<br>CTGACCTTAGGTGATCCGCCT<br>GCCTTGGCCTCCCAAAGTGCT<br>GGGATTACAGGTGTAAGCCAC<br>CGTGCCTGGCCCATGTGTTCTT<br>AATTCATACTGTATCATATCTTG<br>TAAATTTGATTTGTGAGGGAAA<br>TTTAAGCTTTCTAAGATGACAT<br>GAATTCATCACATTCTAACTGA<br>TGGCCTGAAGTGGTGAGGAAT<br>GTTACATGATGCAGAAAGTTGA<br>TATCCCTCCGCTTCTTACTCTT<br>TTTTTTTTTCTCCCCCATCATAC<br>AGGTGAATATGACCATCTCCCA<br>GAACAGGCCTTCTATATGGTG<br>GGACCCATTGAAGAAGCTGTG<br>GCAAAAGCTGATAAGCTGGCT<br>GAAGAGCATTCATCGGGTACC<br>CAAGGCGGTGGAGAATTC	CAAGTCCCTGCGGTGTCTTTG<br>CTTGGATCCGAGGGGTCTTTG<br>TCCTCTGTACTGTCTCTCTCCT<br>TGCCCCTAACCCAAAAAGCTTC<br>ATTTTTCTGTGTAGGCTGCACA<br>AGAGCCTTGATTGAAGATATAT<br>TCTTTCTGAACAGTATTTAAGG<br>TTTCCAATAAAATGTACACCCC<br>TCAGAATTTGTCTGATTCTCTT<br>GGTTCTGACAACATAGTCAACA<br>CTGAAGGGTTATGTATTTAATT<br>TTAGTTTTAGAGACACGGTGTC<br>TGGCTGTGTTGCCAAGACTGG<br>TCTCTAACTCCTGGGCTCGAGA<br>TCTCCCACCTCAGTCTCCTGAG<br>TAGCTGGGGCTACAGGTGTAT<br>GTAGTCTCACATCACCAGCACT<br>GTTTTCAACAATTAGATTTTTAG<br>AGTGGCTATAAGAAGCAGTTTC<br>AGCATGAAGTGGGCCATGTAT<br>GTTTGAAATTGGTCCTTAAAAA<br>TAGCCATTCTCTGGATCCCGG<br>GCCCGTCGACTGCAGAGGCCT	GCGATCCGAGTTCAAATCTCG<br>GTGGAACCTTGAAGAGCATTCA<br>TCGTGAGGTTTTAGAGCTAGAA<br>ATAGCAAGTTAAAAT
HIST1H1C (Histone 1)	GACGTTGTAAAACGACGGCCA<br>GTGGGTACCCTGCCGCCGGCT<br>ATGATGTGGAGAAAAACAACAG<br>CCGTATCAAACTTGGTCTCAAG<br>AGCCTGGTGAGCAAGGGCACT<br>CTGGTGCAAACGAAAGGCACC<br>GGTGCTTCTGGCTCCTTTAAAC<br>TCAACAAGAAGGCAGCCTCCG<br>GGGAAGCCAAGCCCAAGGTTA<br>AAAAGGCGGGCGGAACCAAAC<br>CTAAGAAGCCAGTTGGGGCAG<br>CCAAGAAGCCCAAGAAGGCGG<br>CTGGCGGCGCAACTCCGAAGA<br>AGAGCGCTAAGAAAACACCGA<br>AGAAAGCGAAGAAGCCGGCCG<br>CGGCCACTGTAACCAAGAAAG<br>TGGCTAAGAGCCCAAAGAAGG<br>CCAAGGTTGCGAAGCCCAAGA<br>AAGCTGCCAAAAGTGCTGCTAA<br>GGCTGTGAAGCCCAAGGCCGC<br>TAAGCCCAAGGTTGTCAAGCCT<br>AAGAAA GCAGCA<br>CCTAAGAAGAAAGGTACCCAA<br>GGCGGTGGAGAATTC	CAAGTCCCTGCGGTGTCTTTG<br>CTTGGATCCAGGCGGCGCCCA<br>AGAAGAAATAGGCGAACGCCT<br>ACTTCTAAAACCCAAAAGGCTC<br>TTTTCAGAGCCACCACTGATCT<br>CAATAAAAGAGCTGGATAATTT<br>CTTTACTATCTGCCTTTTCTTGT<br>TCTGCCCTGTTACTTAAGGTTA<br>GTCGTATGGGAGTTACTGAGG<br>TATCAGACGAATTGGGTGACG<br>GGGTTGGAGAGTGGCCGTGGT<br>GAGGTTACAGCATTTAAACCTT<br>TATTGCGGCTTCTAGGTCCCTG<br>ACCGGAGGCTTTTCTCGCTGG<br>CGGATGGTTTTGGGATGGCAG<br>TCCCGCCCCAGGCCTGTGAAC<br>GGCAGAAAAGACCGCAAAACA<br>AGAGCCAGTTTCTTAGTCTAAA<br>GGGATGTCCGGATTGGACTAA<br>AAAATTTTCAAAAGTCCCGCCC<br>TGCTCCCGGGTTGGTCCGTTC<br>TTCTAGTACATGACTTTCAGGA<br>TCCCGGGCCCGTCGACTGCAG<br>AGGCCT	GCGATCCGAGTTCAAATCTCG<br>GTGGAACCTGGTTGTCAAGCC<br>TAAGAAGGGTTTTAGAGCTAGA<br>AATAGCAAGTTAAAAT

H3F3B (Histone 3.3)	GACGTTGTAAAACGACGGCCA<br>GTGGGTACCCGGCCTTATCTT<br>CGGGGCGTCTTTCTTAGGTGA<br>AAGAAAATGGCCCGAACCAAG<br>CAGACTGCTCGTAAGTCCACC<br>GGTGGGAAAGCCCCCCGCAAA<br>CAGCTGGCCACGAAAGCCGCC<br>AGGAAAAGCGCTCCCTCTACC<br>GGCGGGGTGAAGAAGCCTCAT<br>CGCTACAGGTAGGTCGGGCGG<br>GGGAACAATGGCCCGGCGGTG<br>GCCGGCTTTGTGCGGCAGCGT<br>CCGCTCACTCCTCCCCTGCTC<br>GCTGCAGGCCCGGGACCGTG<br>GCGCTTCGAGAGATTCGTCGT<br>TATCAGAAGTCGACCGAGCTG<br>CTCATCCGGAAGCTGCCCTTC<br>CAGAGGTTGGTGAGGGAGATC<br>GCGCAGGATTTCAAAACCGAC<br>CTGAGGTTTCAGAGCGCAGCC<br>ATCGGTGCGCTGCAGGTAAGA<br>CAAAGGCCTGGAGCCGGGGG<br>AGGGCTGGGCGGTTTCCGCTC<br>CCCGAGTGGGATTAATAGTGC<br>GGCTCTCGTCCTCAACAGGAG<br>GCTAGCGAAGCGTACCTGGTG<br>GGTCTGTTCGAAGATACCAACC<br>TGTGTGCCATCCACGCTAAGA<br>GAGTCACCATCATGCCCAAAG<br>ACATCCAGTTGGCTCGCCGGA<br>TAAGAGGAGAACGGGCGGGTA<br>CCCAAGGCGGTGGAGAATTC	CAAGTCCCTGCGGTGTCTTTG<br>CTTGGATCCACGGGGAGAGAG<br>AGCTTAAGTGAAGGCAGTTTTT<br>ATGGCGTTTTGTAGTAAATTCT<br>GTAAAATACTTTGGTTTAATTTG<br>TGACTTTTTTTGTAAGAAATTGT<br>TTATAATATGTTGCATTTGTACT<br>TAAGTCATTCCATCTTTCACTC<br>AGGATGAATGCGAAAAGTGAC<br>TGTTCACAGACCTCAGTGATGT<br>GAGCACTGTTGCTCAGGAGTG<br>ACAAGTTGCTAATATGCAGAAG<br>GGATGGGTGATACTTCTTGCTT<br>CTCATGATGCATGTTTCTGTAT<br>GTTAATGACTTGTTGGGTAGCT<br>ATTAAGGTACTAGAGTTGATAA<br>ATGTGTACAGGGTCCTTTTGCA<br>ATAAAACTGGTTATGACTTGAT<br>CCAAGTGTTTAACAATTGGGGC<br>TGTTAAGTCTGACCATACATCA<br>CTGTGATAGAATGTGGGCTTTT<br>TCAAGGGTGAAGATACAAGTCT<br>TAACCACAGTGTAACTTACAGT<br>TTCCTTTAAAAAAGGATCCCGG<br>GCCCGTCGACTGCAGAGGCCT	GCGATCCGAGTTCAAATCTCG<br>GTGGAACCTCAGTTGGCTCGC<br>CGGATACGGTTTTAGAGCTAGA<br>AATAGCAAGTTAAAAT
TUBB (Tubulin)	GACGTTGTAAAACGACGGCCA<br>GTGGGTACCAGAACATGATGG<br>CTGCCTGTGACCCCCGCCACG<br>GCCGATACCTCACCGTGGCTG<br>CTGTCTTCCGTGGTCGGATGT<br>CCATGAAGGAGGTCGATGAGC<br>AGATGCTTAACGTGCAGAACAA<br>GAACAGCAGCTACTTTGTGGAA<br>TGGATCCCCAACAATGTCAAGA<br>CAGCCGTCTGTGACATCCCAC<br>CTCGTGGCCTCAAGATGGCAG<br>TCACCTTCATTGGCAATAGCAC<br>AGCCATCCAGGAGCTCTTCAA<br>GCGCATCTCGGAGCAGTTCAC<br>TGCCATGTTCCGCCGGAAGGC<br>CTTCCTCCACTGGTACACAGG<br>CGAGGGCATGGACGAGATGGA<br>GTTCACCGAGGCTGAGAGCAA<br>CATGAACGACCTCGTCTCTGA<br>GTATCAGCAGTACCAGGATGC<br>CACCGCAGAAGAGGAGGAGGA<br>TTTCGGTGAGGAGGCCGAAGA<br>GGAGGGTACCCAAGGCGGTG<br>GAGAATTC	CAAGTCCCTGCGGTGTCTTTG<br>CTTGGATCCCTAAGGCAGAGC<br>CCCCATCACCTCAGGCTTCTCA<br>GTTCCCTTAGCCGTCTTACTCA<br>ACTGCCCCTTTCCTCTCCCTCA<br>GAATTTGTGTTTGCTGCCTCTA<br>TCTTGTTTTTTGTTTTTTCTTCT<br>GGGGGGGGTCTAGAACAGTGC<br>CTGGCACATAGTAGGCGCTCA<br>ATAAATACTTGTTTGTTGAATGT<br>CTCCTCTCTCTTTCCACTCTGG<br>GAAACCTAGGTTTCTGCCATTC<br>TGGGTGACCCTGTATTTCTTTC<br>TGGTGCCCATTCCATTTGTCCA<br>GTTAATACTTCCTCTTAAAAATC<br>TCCAAGAAGCTGGGTCTCCAG<br>ATCCCATTTAGAACCAACCAGG<br>TGCTGAAAACACATGTAGATAA<br>TGGCCATCATCCTAAGCCCAAA<br>GTAGAAAATGGTAGAAGGTAGT<br>GGGTAGAAGTCACTATATAAGG<br>AAGGGGATGGGGATCCCGGG<br>CCCGTCGACTGCAGAGGCCT	GCGATCCGAGTTCAAATCTCG<br>GTGGAACCTGAGGCCGAAGAG<br>GAGGCCTAGTTTTAGAGCTAGA<br>AATAGCAAGTTAAAAT

NFE2L2 (Nuclear factor (erythroid-derived 2)-like 2 or NRF2)	GACGTTGTAAAACGACGGCCAGTGGGTACCTTAGGGCAAAAGCTCTCCATATCCCATTCCCTGTAGAAAAAATCATTAACCTCCCTGTTGTTGACTTCAACGAAATGATGTCCAAAGAGCAGTTCAATGAAGCTCAACTTGCATTAATTCGGGATATACGTAGGAGGGGTAAGAATAAAGTGGCTGCTCAGAATTGCAGAAAAAGAAAACTGGAAAATATAGTAGAACTAGAGCAAGATTTAGATCATTTGAAAGATGAAAAAGAAAAATTGCTCAAAGAAAAAGGAGAAAATGACAAAAGCCTTCACCTACTGAAAAAACAACTCAGCACCTTATATCTCGAAGTTTTCAGCATGCTACGTGATGAAGATGGAAAACCTTATTCTCCTAGTGAATACTCCCTGCAGCAAACAAGAGATGGCAATGTTTTCCTTGTTCCCAAAAGTAAGAAGCCAGATGTTAAGAAAAACGGTACCCAAGGCGGTGGAGAATTC	CAAGTCCCTGCGGTGTCTTTGCTTGGATCCAGGAGGATTTGACCTTTTCTGAGCTAGTTTTTTTGTACTATTATACTAAAAGCTCCTACTGTGATGTGAAATGCTCATACTTTATAAGTAATTCTATGCAAAATCATAGCCAAAACTAGTATAGAAAATAATACGAAACTTTAAAAAGCATTGGAGTGTCAGTATGTTGAATCAGTAGTTTCACTTTAACTGTAAACAATTTCTTAGGACACCATTTGGGCTAGTTTCTGTGTAAGTGTAAATACTACAAAAACTTATTTATACTGTTCTTATGTCATTTGTTATATTCATAGATTTATATGATGATATGACATCTGGCTAAAAAGAAATTATTGCAAAACTAACCACTATGTACTTTTTTATAAATACTGTATGGACAAAAAATGGCATTTTTTATATTAAATTGTTTAGGATCCCGGGCCCGTCGACTGCAGAGGCCT	GCGATCCGAGTTCAAATCTCGGTGGAACCTTAAGAAAAACTAGATTTAGGGTTTTAGAGCTAGAAATAGCAAGTTAAAAT
SQSTM1 (p62 protein)	GACGTTGTAAAACGACGGCCAGTGGGTACCTATTTCAGTGTCCATTGATGGTTCTGCTTACACACCACCTGGCTGCCTGGTGTCGCAGTGGCAGAGTTGAGCAGTGTGAAAAAGACTGCTTGGCCCTTTACAGGGAAAGCAGGTCCACTGTGGCCTGTGAGGACGAGAGCTCTGGGCAGGCTCGGACACTGGCAGACCCTGGTCCTGGCTGGCCAAGGCAGCAGGGTATGTGTTTCGGGTCACTCACAGGGCTCAGCACCACTCCTCATGGCTTCCTTACTGTTTCGGCAGAGGCTGACCCGCGGCTGATTGAGTCCCTCTCCCAGATGCTGTCCATGGGCTTCTCTGATGAAGGCGGCTGGCTCACCAGGCTCCTGCAGACCAAGAACTATGACATCGGAGCGGCTCTGGACACCATCCAATACAGCAAGCACCCCCCACCGCTTGGTACCCAAGGCGGTGGAGAATTC	CAAGTCCCTGCGGTGTCTTTGCTTGGATCCAGTATTCAAAGCATCCCCCGCCGTTGTGACCACTTTTGCCCACCTCTTCTGCGTGCCCCTCTTCTGTCTCATAGTTGTGTTAAGCTTGCGTAGAATTGCAGGTCTCTGTACGGGCCAGTTTCTCTGCCTTCTTCCAGGATCAGGGGTTAGGGTGCAAGAAGCCATTTAGGGCAGCAAAACAAGTGACATGAAGGGAGGGTCCCTGTGTGTGTGTGTGCTGATGTTTCCTGGGTGCCCTGGCTCCTTGCAGCAGGGCTGGGCCTGCGAGACCCAAGGCTCACTGCAGCGCGCTCCTGACCCCTCCCTGCAGGGGCTACGTTAGCAGCCCAGCACATAGCTTGCCTAATGGCTTTCACTTTCTCTTTTGTTTTAAATGACTCATAGGTCCCTGACATTTAGTTGATTATTTTCTGCTACGGATCCCGGGCCCGTCGACTGCAGAGGCCT	GCGATCCGAGTTCAAATCTCGGTGGAACCTGGGATGCTTTGAATACTGGAGTTTTAGAGCTAGAAATAGCAAGTTAAAAT
PARP1 (Poly [ADP-ribose] polymerase 1)	GACGTTGTAAAACGACGGCCAGTGGGTACCGGCAGACAAGGATTAGAGGCTGTCCTGTAGTGTGTCCCATGGTGACTGTTTCCCTTCTGTGGTCCCTCCCTGTGCATAGCCTGGCTTACAGGGTATGAGCCTTCCCCCAGTTTCCAGAGGATGATCTCCTCTCCTCAGTCTGCCTGAAGAAGACTTAGAGTAACTTTCAGGCTGGCATTGAGCATCCTGCCAGCCCCGGGGAGATGAGGCAACCCAGCCCCATGAAGAGGCCTTAGAGTGACTTTCAGGCTGGCACTGAGCGTCCTGCCAGCCTGGGGGAGATGAGGCACATGTACATACCCTCTGTTGTATGGCTGTTGGCTCCTTAACAAGCTTCCCCTCAGGTACATTGTCTATGATATTGCTCAGGTAAATCTGAAGTATCTGCTGAAACTGAAATTCAATTTTAAGACCTCCTTATGGGGTACCCAAGGCGGTGGAGAATTC	CAAGTCCCTGCGGTGTCTTTGCTTGGATCCCTGTGGTAATTGGGAGAGGTAGCCGAGTCACACCCGGTGGCTCTGGTATGAATTCACCCGAAGCGCTTCTGCACCAACTCACCTGGCCGCTAAGTTGCTGATGGGTAGTACCTGTACTAAACCACCTCAGAAAGGATTTTACAGAAACGTGTTAAAGGTTTTCTCTAACTTCTCAAGTCCCTTGTTTTGTGTTGTGTCTGTGGGGAGGGGTTGTTTTGGGGTTGTTTTTGTTTTTTCTTGCCAGGTAGATAAAACTGACATAGAGAAAAGGCTGGAGAGAGATTCTGTTGCATAGACTAGTCCTATGGAAAAAACCAAGCTTCGTTAGAATGTCTGCCTTACTGGTTTCCCCAGGGAAGGAAAAATACACTTCCACCCTTTTTTCTAAGTGTTCGTCTTTAGTTTTGATTTTGGAAAGGATCCCGGGCCCGTCGACTGCAGAGGCCT	GCGATCCGAGTTCAAATCTCGGTGGAACCTTCAATTTTAAGACCTCCCTGGTTTTAGAGCTAGAAATAGCAAGTTAAAAT
