# Supplementary material for: Multiplex Gene Tagging with CRISPR-Cas9 for Live-Cell Microscopy and Application to Study the Role of SARS-CoV-2 Proteins in Autophagy, Mitochondrial Dynamics, and Cell Growth
Source: CRISPR J. 2021 Dec 16;4(6):854–71. doi: 10.1089/crispr.2021.0041 (PMC8742308; doi:10.1089/crispr.2021.0041)
Supplement: Supplemental data [file Suppl_FigS1.pdf]

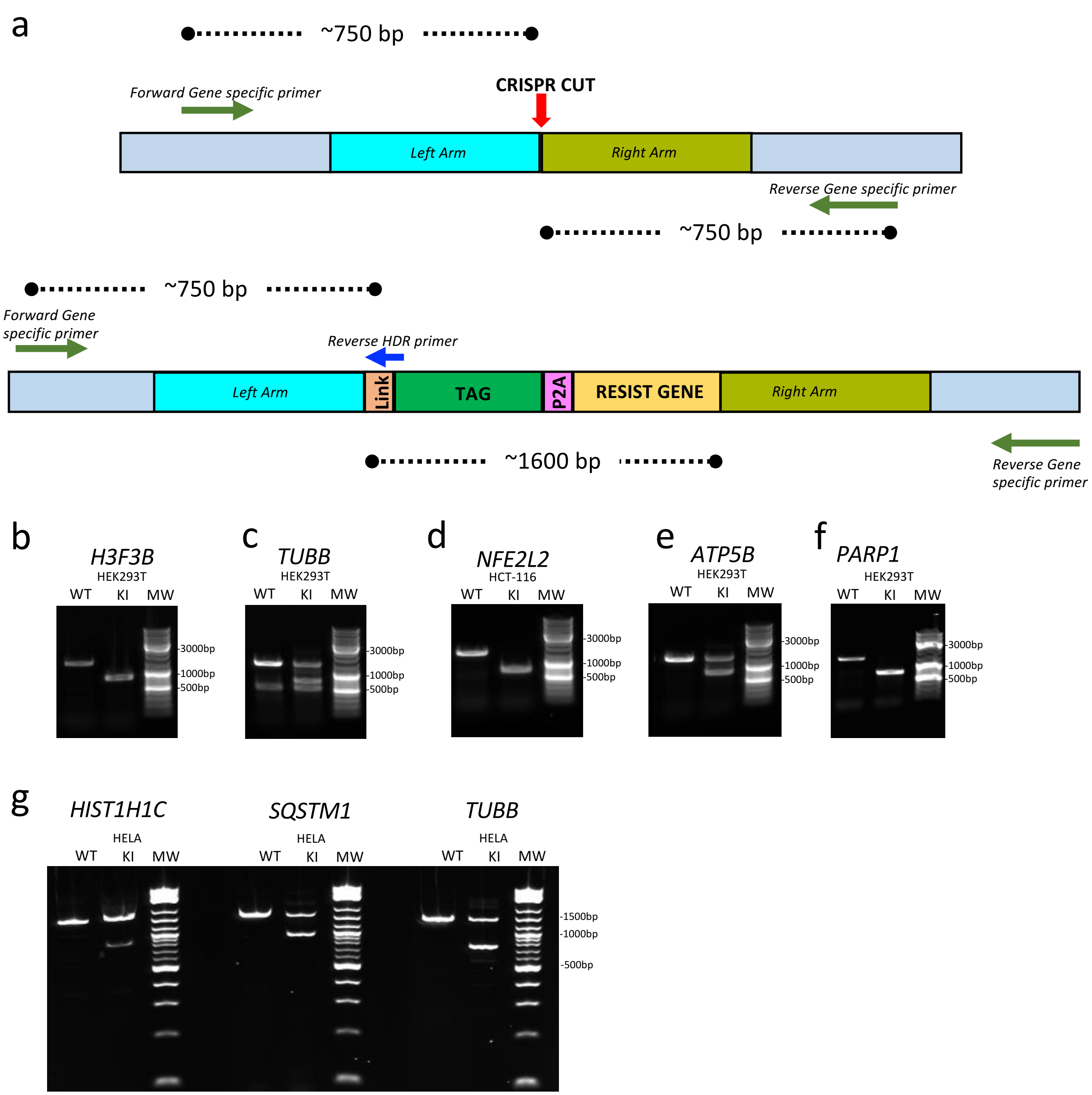

Figure S1. Validation of genomic insertion of the labeling tags. (a) The genomic insertion was verified by PCR using a set of three primers. Two primers (green, forward and reverse) annealed around the desired CRISPR cutting site and generated a PCR product of ~1,500 bp if the region was not modified (upper panel). The third primer (reverse, in blue) produced a PCR product of ~750 bp only if the labeling tag was inserted at the desired location (lower panel). (b-g) The pattern of PCR products in wild-type and modified cells allowed us to determine the absence of modification or the presence of heterozygous or homozygous labeling. The correct targeting of the genes described in this work was validated as follows: (b) HEK293T cells with H3.3 labeled with mRuby3 (homozygous), (c) HEK293T cells with β tubulin labeled with mClover3 (Heterozygous), (d) HCT116 cells with NRF2 labeled with NanoLuc (homozygous), (e) HEK293T cells with ATP5B labeled with mTagBFP2 (heterozygous), (f) HEK293T cells with PARP1 labeled with mClover3 (homozygous), (g) HELA cells with multiplexing labeling of HIST1H1C with mTagBFP2 (Heterozygous), SQSTM1 with mRuby3 (Heterozygous) and TUBB with mClover3 (Heterozygous).
