# Supplementary material for: Multiplex Gene Tagging with CRISPR-Cas9 for Live-Cell Microscopy and Application to Study the Role of SARS-CoV-2 Proteins in Autophagy, Mitochondrial Dynamics, and Cell Growth
Source: CRISPR J. 2021 Dec 16;4(6):854–71. doi: 10.1089/crispr.2021.0041 (PMC8742308; doi:10.1089/crispr.2021.0041)
Supplement: Supplemental data [file Suppl_TableS2.pdf]

Supplementary Table 2. Sequences of primers used in this work.

Genes		Sequences (5'-3')
ATP5B	Forward	TGCTGTGGTCCCATTCCAACA
	Reverse	ACGAGTTTTTCCCAATATGCCCTTC
HIST1H1C	Forward	CCAGAGCACCAATCAGAGCG
	Reverse	CCTGTCAGTTTAGCGGAAGGC
H3F3B	Forward	CGCAGCCTGAGTCATTAGGGG
	Reverse	TGCAAGGTATAAATGCGCATAGCA
TUBB	Forward	GCACTCTGAAGCTGACCACACCA
	Reverse	CCTCCCAACCCCCTTGATCCCTT
NFE2L2	Forward	GTGCCCCTGGAAGTGTCAAACA
	Reverse	GTGGGCGTATGTCTACTGATGGAA
SQSTM1	Forward	TCTTTGCATCATCATAGCTTAGCATCT
	Reverse	GTCATTGGTTAAAGTGCTGATGCC
PARP1	Forward	CCCTGGAAGCTTTGCCACATC
	Reverse	AGCCCTTGGGTAAGTATATTTGTGG
Universal Reverse Link	Reverse	ATGAATTCTCCACCGCCTTG
