# Supplementary material for: Multiplex Gene Tagging with CRISPR-Cas9 for Live-Cell Microscopy and Application to Study the Role of SARS-CoV-2 Proteins in Autophagy, Mitochondrial Dynamics, and Cell Growth
Source: CRISPR J. 2021 Dec 16;4(6):854–71. doi: 10.1089/crispr.2021.0041 (PMC8742308; doi:10.1089/crispr.2021.0041)
Supplement: Supplemental data [file Suppl_FigS2.pdf]

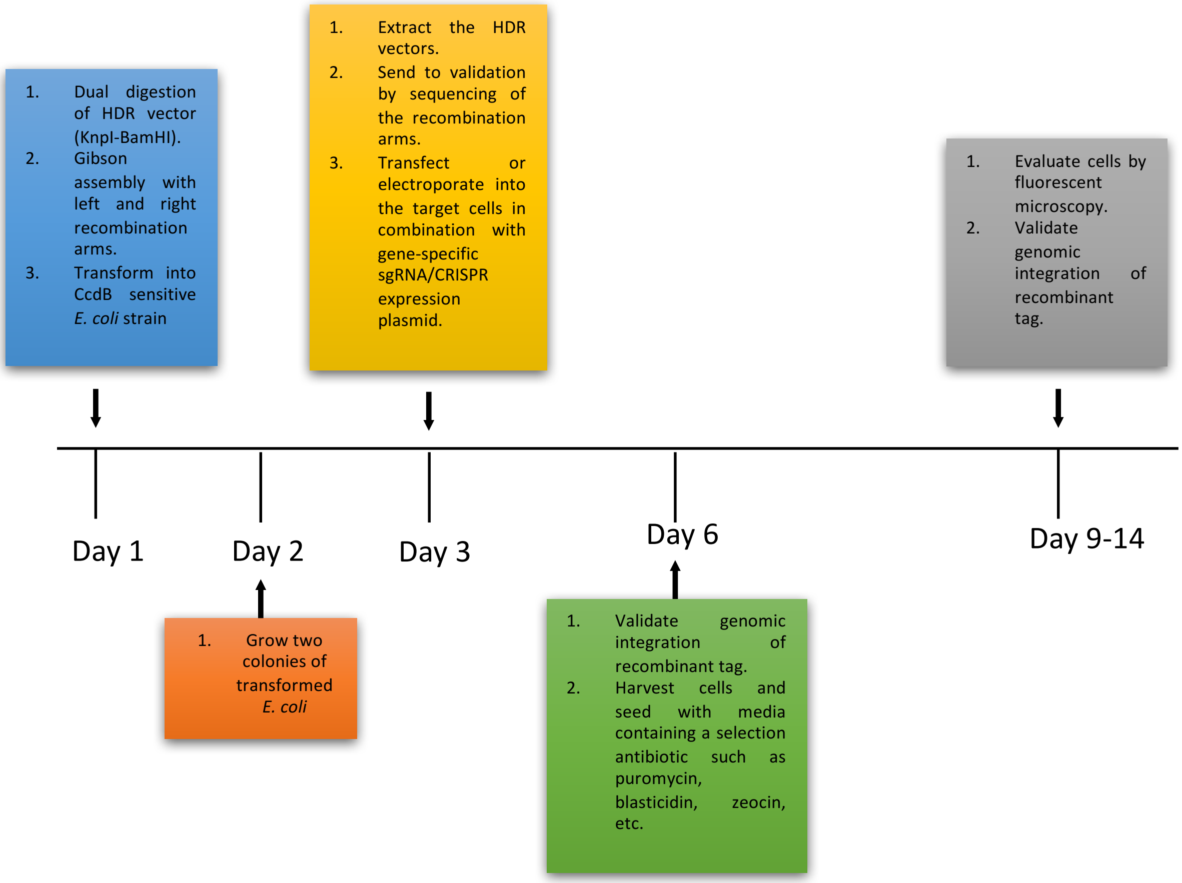

Figure S2. Diagram of the process for developing homologous recombination vectors and selection of modified cell lines with the FAST-HDR system.
