# Supplementary material for: Multiplex Gene Tagging with CRISPR-Cas9 for Live-Cell Microscopy and Application to Study the Role of SARS-CoV-2 Proteins in Autophagy, Mitochondrial Dynamics, and Cell Growth
Source: CRISPR J. 2021 Dec 16;4(6):854–71. doi: 10.1089/crispr.2021.0041 (PMC8742308; doi:10.1089/crispr.2021.0041)
Supplement: Supplemental data [file Suppl_FigS3.pdf]

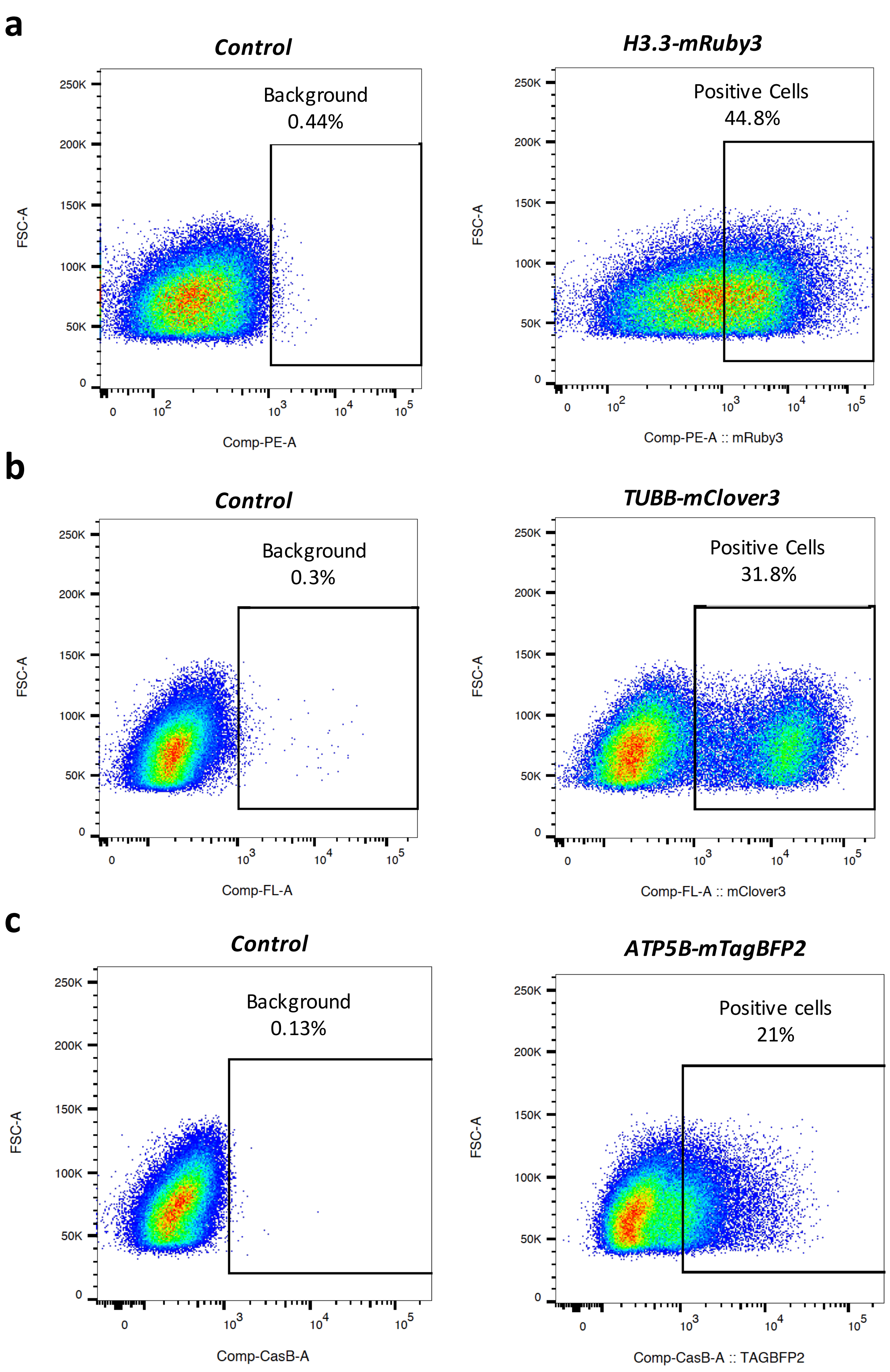

Figure S3. Flow cytometry analysis to detect HEK293T cells with a single genetically tagged gene before antibiotic selection. Detection of fluorescent cells after seventy hours of transfection with two plasmids (a plasmid encoding CRISPR-Cas9 with a gene specific sgRNA and a FAST-HDR donor template plasmid). Unmodified HEK293T cells were used as control. (a) C-term tagging of Histone 3.3 with mRuby3. (b) C-term tagging of β Tubulin with mClover3. (c) C-term tagging of ATP5B with mTagBFP2. This figure is representative of three independent experiments.
