# Supplementary material for: Multiplex Gene Tagging with CRISPR-Cas9 for Live-Cell Microscopy and Application to Study the Role of SARS-CoV-2 Proteins in Autophagy, Mitochondrial Dynamics, and Cell Growth
Source: CRISPR J. 2021 Dec 16;4(6):854–71. doi: 10.1089/crispr.2021.0041 (PMC8742308; doi:10.1089/crispr.2021.0041)
Supplement: Supplemental data [file Suppl_FigS5.pdf]

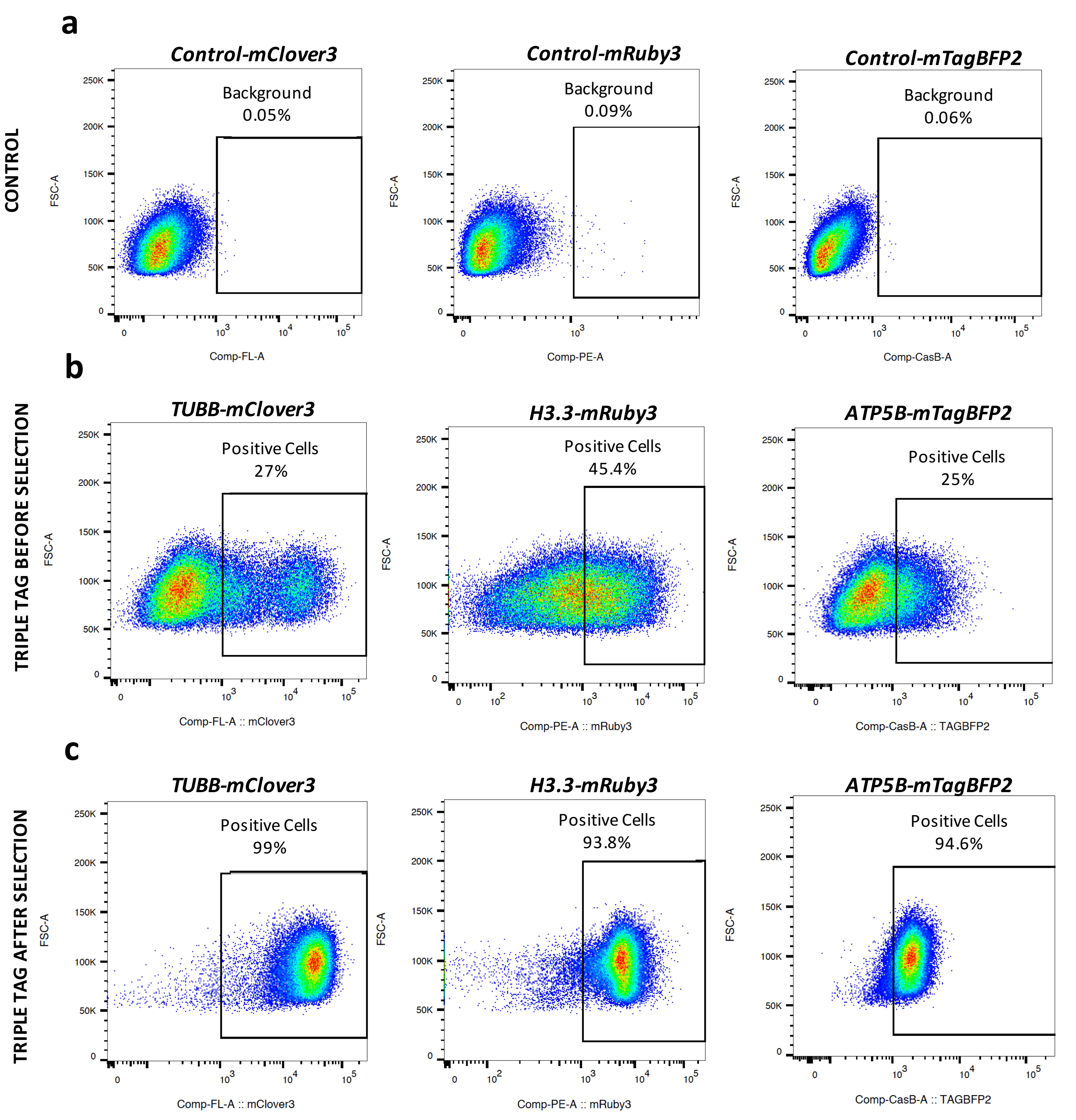

Figure S5. Flow cytometry analysis to detect HEK293T cells with three endogenous tagged genes before and after antibiotic selection. HEK293T cells were transfected with six plasmids (three plasmids encoding CRISPR-Cas9 with a gene specific sgRNA and three FAST-HDR donor template plasmids) to induce gene tagging with three fluorescent proteins. (a) Unmodified HEK293T cells were used as control. (b) Detection of HEK293T cells with C-term tagging of Histone 3.3 with mRuby3, β Tubulin with mClover3 and ATP5B with mTagBFP2 seventy hours after transfection and before starting antibiotic selection. (c) Detection of HEK293T cells with C-term tagging of Histone 3.3 with mRuby3, β Tubulin with mClover3 and ATP5B with mTagBFP2 ten days after starting antibiotic selection. This figure is representative of three independent experiments.
