# Supplementary material for: Multiplex Gene Tagging with CRISPR-Cas9 for Live-Cell Microscopy and Application to Study the Role of SARS-CoV-2 Proteins in Autophagy, Mitochondrial Dynamics, and Cell Growth
Source: CRISPR J. 2021 Dec 16;4(6):854–71. doi: 10.1089/crispr.2021.0041 (PMC8742308; doi:10.1089/crispr.2021.0041)
Supplement: Supplemental data [file Suppl_FigS6.pdf]

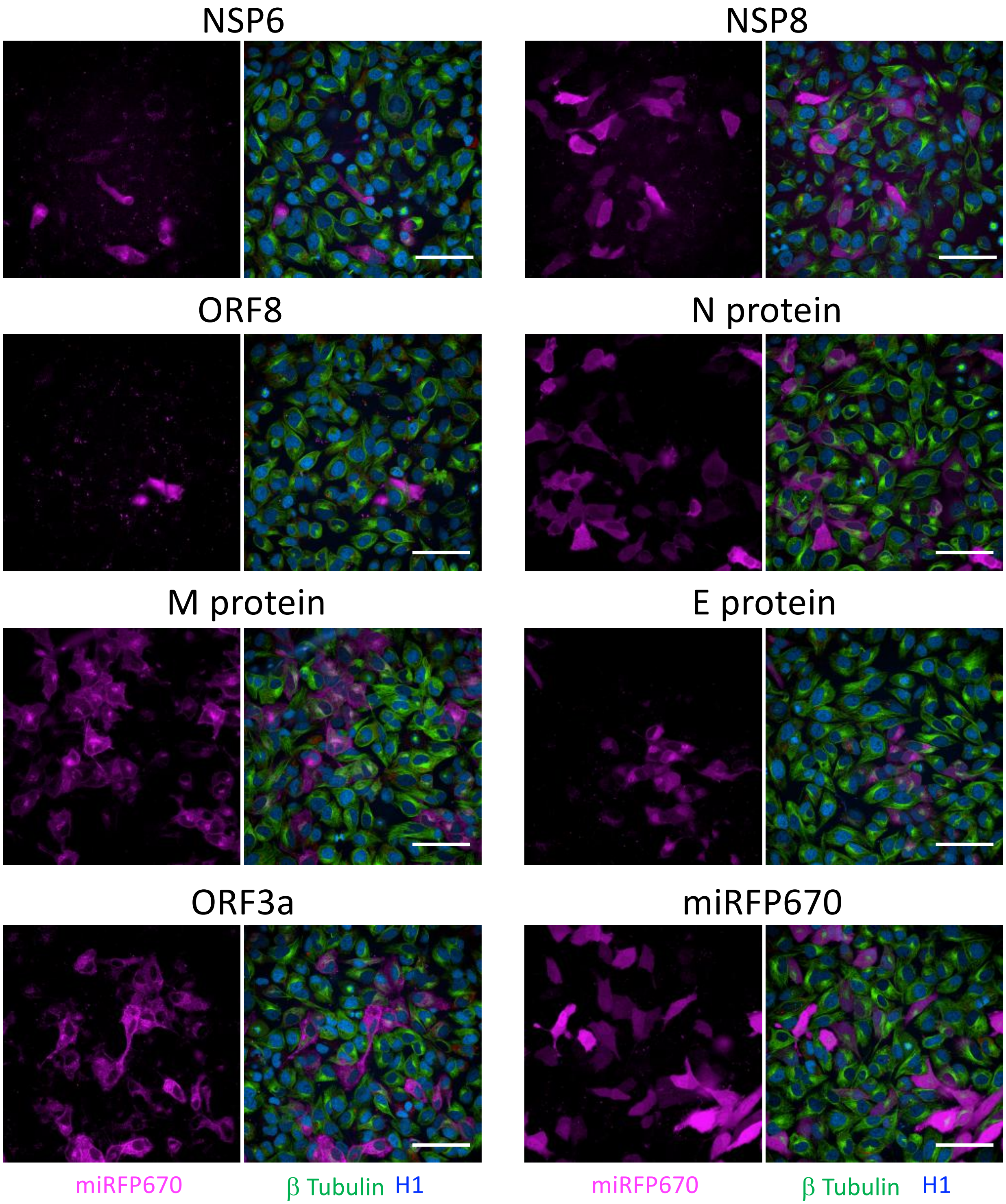

Figure S6. Overexpression of SARS-coV-2 proteins in a multiplex gene-tagged HeLa cell line. Seven SARS-coV-2 proteins with a c-terminal miRFP670 tag, and miRFP670 protein only as a control were overexpressed by transient transfection and evaluated after 24 hours. The overexpression was done in HeLa cells containing endogenous tagged beta Tubulin (mClover3), Histone 1 (mtagBFP2), and P62/SQSTM1 (mRuby3); this last channel was not included in the image to facilitate visualization. Bar equals 50 μm.
